# Dandelion uses the single-cell adaptive immune receptor repertoire to explore lymphocyte developmental origins

**DOI:** 10.1038/s41587-023-01734-7

**Published:** 2023-04-13

**Authors:** Chenqu Suo, Krzysztof Polanski, Emma Dann, Rik G. H. Lindeboom, Roser Vilarrasa-Blasi, Roser Vento-Tormo, Muzlifah Haniffa, Kerstin B. Meyer, Lisa M. Dratva, Zewen Kelvin Tuong, Menna R. Clatworthy, Sarah A. Teichmann

**Affiliations:** 1 Wellcome Sanger Institute, Wellcome Genome Campus, Cambridge, UK; 2 Department of Paediatrics, Cambridge University Hospitals, Cambridge, UK; 3 Biosciences Institute, Newcastle University, Newcastle upon Tyne, UK; 4 Department of Dermatology and NIHR Newcastle Biomedical Research Centre, Newcastle upon Tyne Hospitals NHS Foundation Trust, Newcastle upon Tyne, UK; 5 Molecular Immunity Unit, Department of Medicine, University of Cambridge, Cambridge, UK; 6 Frazer Institute, Faculty of Medicine, The University of Queensland, Brisbane, Queensland Australia; 7 Theory of Condensed Matter, Cavendish Laboratory, Department of Physics, University of Cambridge, Cambridge, UK; 8 Present Address: Ian Frazer Centre for Children’s Immunotherapy Research, Child Health Research Centre, Faculty of Medicine, The University of Queensland, Brisbane, Queensland Australia

**Keywords:** Computational biology and bioinformatics, Immunology

## Abstract

Assessment of single-cell gene expression (single-cell RNA sequencing) and adaptive immune receptor (AIR) sequencing (scVDJ-seq) has been invaluable in studying lymphocyte biology. Here we introduce Dandelion, a computational pipeline for scVDJ-seq analysis. It enables the application of standard V(D)J analysis workflows to single-cell datasets, delivering improved V(D)J contig annotation and the identification of nonproductive and partially spliced contigs. We devised a strategy to create an AIR feature space that can be used for both differential V(D)J usage analysis and pseudotime trajectory inference. The application of Dandelion improved the alignment of human thymic development trajectories of double-positive T cells to mature single-positive CD4/CD8 T cells, generating predictions of factors regulating lineage commitment. Dandelion analysis of other cell compartments provided insights into the origins of human B1 cells and ILC/NK cell development, illustrating the power of our approach. Dandelion is available at https://www.github.com/zktuong/dandelion.

## Abstract (web summary)

A computational pipeline enables differential V(D)J usage analysis and pseudotime trajectory inference from single-cell AIR sequencing.

## Main

Single-cell genomics has advanced our understanding of human immunology^1,2^. Paired adaptive immune receptor (AIR) sequencing with mRNA expression in the same cell allows for direct linkage of AIR repertoire with cellular phenotypes, which is a powerful way to understand lymphocyte development and function^3–6^.

**Extended Data Fig. 1:**
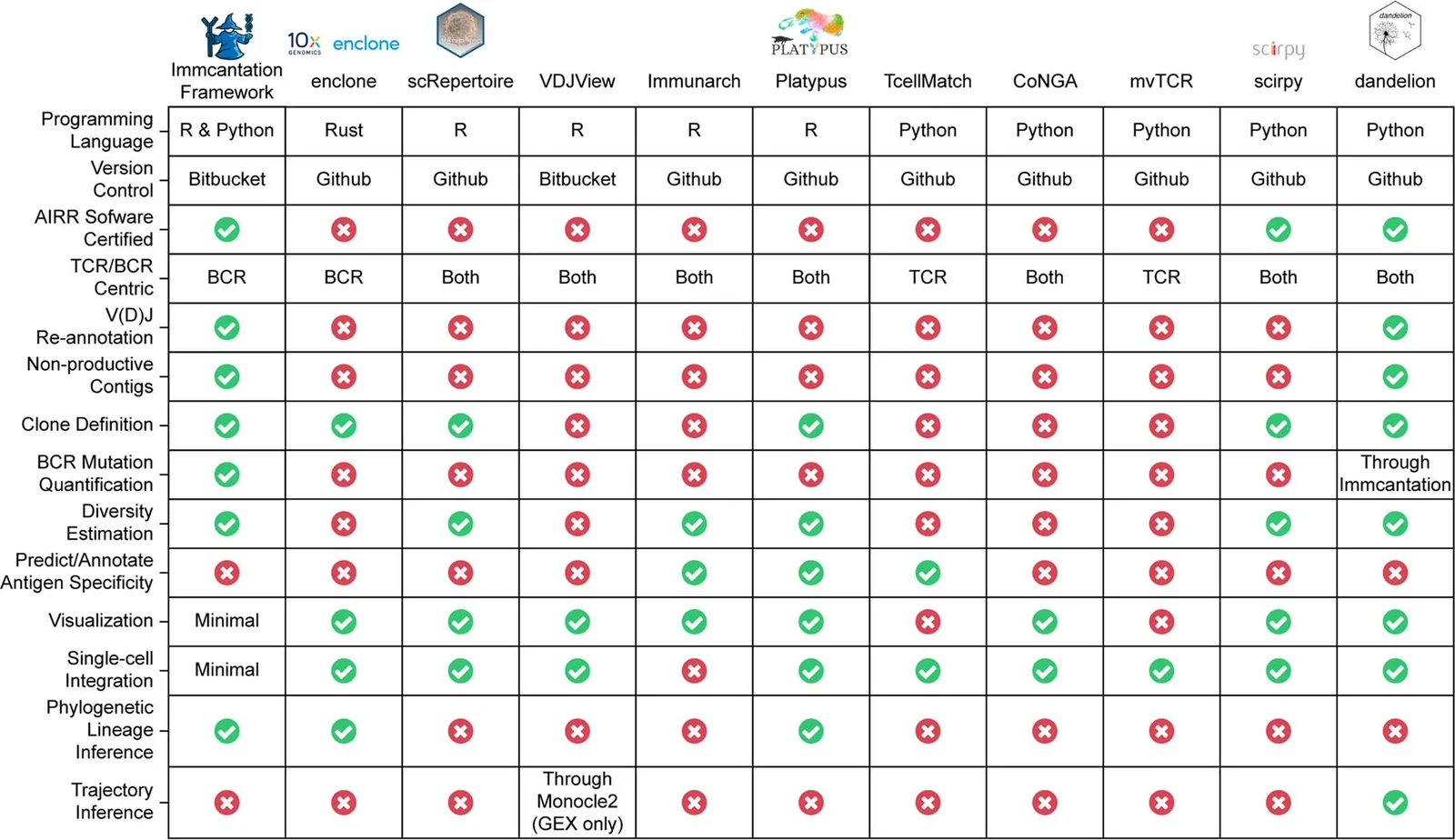
List of features included in AIR repertoire analysis pipelines. A table outlining the features of a non-exhaustive list of other methods compared to *Dandelion*. Handling of non-productive contigs (with or without V gene annotation) is not common across the various software packages. While the *Immcantation* workflow is capable of handling the data, contigs without V genes are typically diverted to a ‘failed’ file but can be retrieved separately. The output from *Dandelion* is compatible with any AIRR-compliant software for example *Dandelion* output can be passed to *Immcantation* to perform phylogenetic lineage inference.

Multi-omics analysis has enabled the study of cellular biology across data modalities at an unprecedented resolution. This includes the integration of paired single-cell RNA sequencing (scRNA-seq) and assay for transposase-accessible chromatin with high-throughput sequencing data or cellular indexing of transcriptomes and epitopes by sequencing data^7,8^. However, unlike these modalities, which largely consist of continuous data, AIR data consist of a mixture of categorical and continuous data, posing additional challenges for integration. This includes annotations of variable (V), diversity (D) and joining (J) genes, which are recombined and selected during B/T cell development^9^. The Adaptive Immune Receptor Repertoire (AIRR) community was formed in 2015 to help address challenges related to AIR data analysis^10–12^. This has led to the standardization of repertoire data representation across AIR analysis domains. There are established packages that can deal with single-cell AIR repertoire data and they provide a variety of methods for downstream analyses (nonexhaustive list of popular tools is shown in Extended Data Fig. 1). The functions include re-annotation of AIR genes, quality control checks, matching contigs to cells, clonotype definition, mutation quantification, diversity estimation and many more (Extended Data Fig. 1). Single-cell AIR software are often designed to interact with a companion single-cell gene expression software, for example, scirpy^13^ with scanpy^14^ and scRepertoire^15^ with Seurat^16^, providing valuable analysis and visualization options. There are also tools for predicting antigen specificity of T cell receptors (TCRs; for example, TcellMatch^17^), annotating TCRs with epitopes (for example, Platypus^18^ and Immunarch^19^) and extraction of significant motifs and motif groups (for example, ALICE^20^). Tools for joint embedding of single-cell gene expression and AIR complementarity-determining region 3 (CDR3) sequences have also been developed (for example, CoNGA^21^ and mvTCR^22^). There remain opportunities for new methods to realize the full potential of paired scRNA-seq and scVDJ-seq data.

To that end, we developed Dandelion, a holistic analysis framework for understanding single-cell lymphocyte biology. It offers a B cell receptor (BCR) and TCR contig annotation pipeline, integrative analysis with single-cell RNA-seq data and a V(D)J feature space for differential V(D)J usage and pseudotime trajectory inference. Here using two immune development datasets, we showcase how Dandelion can improve the alignment of cells along T cell development trajectory and provide insights into human B1 cell origin and innate lymphoid cell (ILC) and natural killer (NK) cell development.

## Results

### Dandelion enables holistic scVDJ-seq analysis

**Fig. 1:**
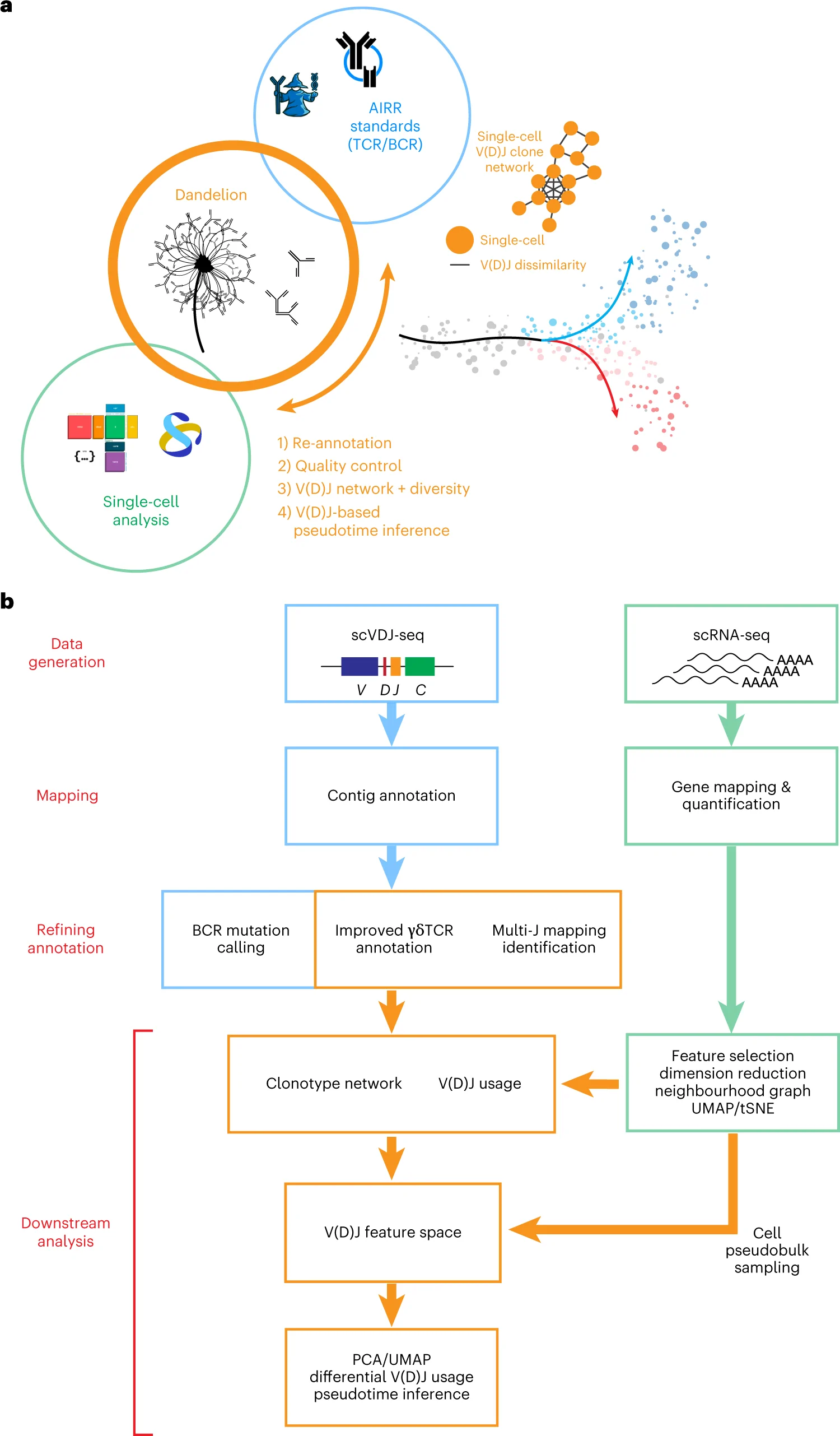
Holistic scVDJ-seq analysis pipeline. **a**, Schematic illustration showing that Dandelion bridges methods from single-cell V(D)J workflows, such as AIRR standards and the single-cell gene expression analysis software, and combines with them additional new methods of its own to create a holistic pipeline for analysis. **b**, Schematic illustration of the Dandelion workflow. Paired single-cell gene expression (scRNA-seq) and AIR repertoire (scVDJ-seq) data are generated, followed by mapping of the sequencing reads. From the mapped results, Dandelion provides refined contig annotations with BCR mutation calling, improved γδTCR mapping and identification of multi-J mapping contigs. It also provides downstream analysis after integration with scRNA-seq results. Apart from allowing the users to explore clonotype networks and V(D)J usage, Dandelion also supports building a V(D)J feature space on pseudobulked cells, that can be used for differential V(D)J usage and pseudotime inference. Additional unique features provided by Dandelion are boxed in orange.

As Dandelion operates on the AIRR data format, it is highly interoperable with existing AIRR tools^13,23^. It can serve as a bridge between the various tools for AIRR analysis and the single-cell software ecosystem, for example, scverse^14,24^ (Fig. 1a). Dandelion has been certified by the AIRR Software Working Group to be AIRR standards compliant.

Dandelion can be used to analyze single-cell BCR, αβTCR and γδTCR data, allowing for mutation calling, improved γδTCR annotation, analysis of productive and nonproductive V(D)J contigs and identification of unspliced *J* gene alignments (Fig. 1b). Dandelion performs quality control checks, clonotype calling and network generation for downstream analyses. It is designed to work with AIRR-formatted input or 10X Genomics’ cellranger vdj output. A main feature of Dandelion is the creation of a ‘V(D)J feature space’ that can be used to perform and visualize differential TCR/BCR usage across cell pseudo-bulks or neighborhoods and infer pseudotime trajectory inference. Extended Data Fig. 1 shows a summary of features in Dandelion and other pipelines. Dandelion was previously applied to a large COVID-19 study^4^, which showcased its network-based repertoire diversity analysis method.

### Dandelion provides a streamlined preprocessing pipeline

For optional re-annotation of contigs, Dandelion expects 10X Genomics’ cellranger vdj output files (for example, all_contig_annotations.csv and all_contig.fasta).

Similar to Change-O^23^, Dandelion re-annotates V(D)J contigs using igblastn^25^ with reference sequences contained in the international ImMunoGeneTics information system (IMGT) database^26^. blastn is also used to check the *D* and *J* genes separately (same settings as igblastn^25^. The additional blastn step allows us to (1) apply an *e*-value cutoff for D/J calls to only retain high confidence calls; (2) identify ‘multi-J mapping’ contigs (see below) and (3) recover contigs without *V* gene calls (removed by igblastn). We packaged this preprocessing workflow into a singularity container to streamline and improve the user experience and avoid the difficulties with setting up the pipeline.

**Fig. 2:**
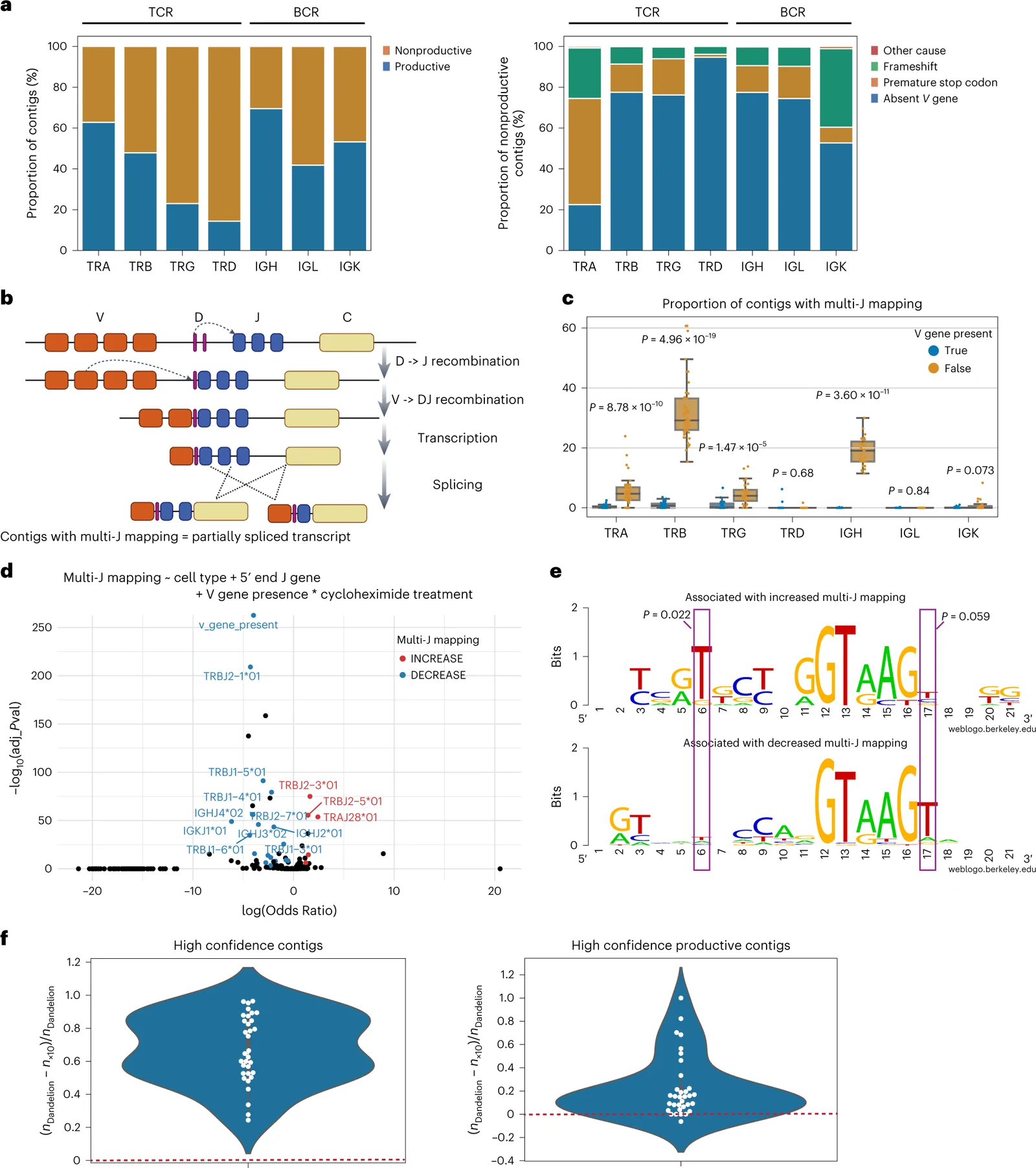
Dandelion offers improved contig annotations. **a**, Left—barplot of proportion of contigs that are productive or nonproductive in each locus. Right—barplot showing the causes of nonproductive contigs in each locus. **b**, Schematic illustration of the V(D)J rearrangement process and the potential cause of multi-J mapping. **c**, Boxplot of the proportion of contigs with multi-J mapping, in the presence (blue) or absence (orange) of *V* genes. Only samples with at least ten contigs are shown. Boxes capture the first to third quartiles and whisks span a further 1.5× interquartile range on each side of the box (two-sided Wilcoxon rank-sum test). The sample sizes of all boxplots from left to right are 51, 46, 52, 55, 26, 29, 20, 29, 33, 27, 33, 29, 34 and 25. **d**, Top—logistic regression formula to explore factors associated with multi-J mapping. Bottom—volcano plot summarizing logistic regression results (ref. ^3^); *y* axis: −log_10_(BH adjusted *P* value); *x* axis: log(odds ratio). Variables that were also significant in our control/cycloheximide-treated PBMC dataset are highlighted in red (associated with increased multi-J mapping) or blue (associated with decreased multi-J mapping). **e**, Sequence logos covering the last 11 and first 10 nucleotides at 3′ ends (position 1–11) and the neighboring intron (position 12–21), respectively, for genes associated with increased (top) or decreased (bottom) multi-J mapping. *J* genes associated with increased multi-J mapping were less likely to have T in position 17 (logistic regression; two-tailed *P* value). ‘GTAAGT’ is a known consensus motif for splicing in position 12–17 that is +1 to +6 in the intron. They were also more likely to have T in position 6 (logistic regression; two-tailed *P* value). **f**, Swarmplots of fraction difference of sc-γδTCR contigs (*n* = 33) annotated by Dandelion versus 10X cellranger vdj (v6.1.2). The red dashed line marks the threshold of 0, above which Dandelion recovers more γδTCR contigs than 10X. Left—all high confidence contigs. Right—high confidence productive contigs. Data for **a**, **c**, **d** (bottom) and **f** were taken from ref. ^3^ and each dot represents a sample.

**Extended Data Fig. 2:**
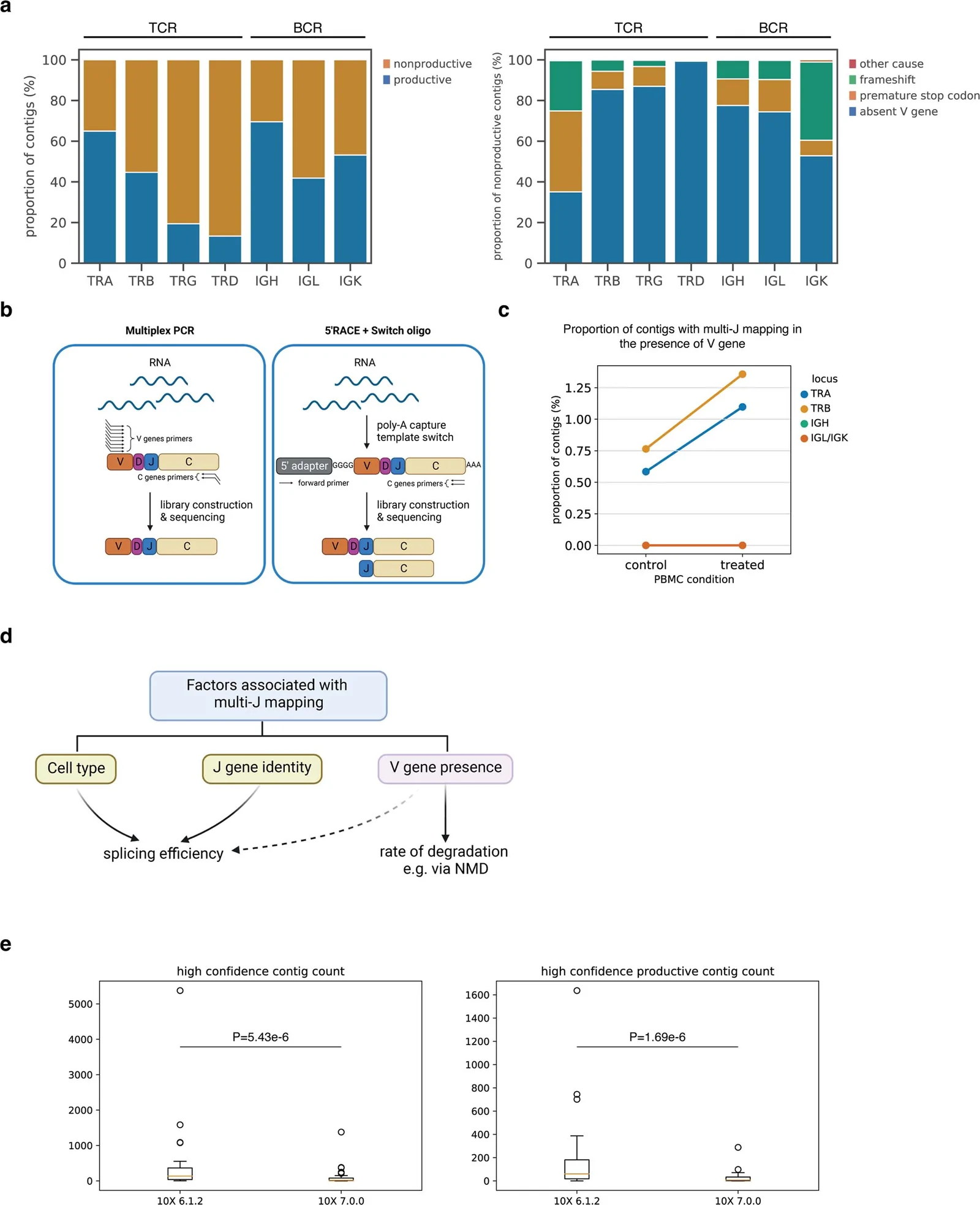
*Dandelion* offers improved contig annotations. **a**, Left: barplot of proportion of contigs that are productive or non-productive in each locus. Right: barplot showing the causes of non-productive contigs in each locus. For both plots, sc-γδTCR, -αβTCR and -BCR data were taken from Suo et al. 2022^3^ excluding thymus samples. **b**, Schematic illustration showing that mRNA without V genes would be captured by 5′RACE + Switch oligo technique but not by multiplex PCR strategy. **c**, Pointplot of proportion of contigs with multi-J mapping in the presence of V gene in control and cycloheximide-treated PBMC samples. Points are colored by locus of TCR/BCR. For both IGH and IGL/IGK, the proportions were 0% in control and treated. **d**, Schematic illustration showing the factors associated with multi-J mapping and the proposed mechanisms. **e**, Boxplots of sc-γδTCR contig counts annotated by 10X *cellranger vdj* v6.1.2 *versus* v7.0.0 using data from n = 33 independent samples from Suo et al. 2022^3^. Left: all high confidence contigs (*P*-value 5.43e-6, r 0.91 in the two-sided Wilcoxon signed-rank test). Right: high confidence productive contigs (*P*-value 1.69e-6, r 0.96 in the two-sided Wilcoxon signed-rank test). Boxes capture the first to third quartiles and whisks span a further 1.5X interquartile range on each side of the box.

Nonproductive contigs do not translate into functional proteins and are often filtered out by other scVDJ-seq analysis pipelines, for example, scirpy^13^, scRepertoire^15^ and Platypus^18^ (Extended Data Fig. 1). In the Immcantation^23^ workflow, nonproductive contigs are preserved and there are specific instructions for filtering or retention during annotation and clone definition steps. Moreover, igblastn is a *V* gene annotation tool^25^ and would filter contigs without *V* gene. We found that a significant proportion of αβTCR, γδTCR and BCR data were nonproductive in fetal human tissues^3^ and the majority were due to absent *V* genes, with the exception of the TRA locus where most were due to presence of premature stop codons (Fig. 2a). This pattern was consistent even after excluding thymic samples to account for developing T cells (Extended Data Fig. 2a). These nonproductive contigs without *V* genes were captured in scVDJ-seq because the rapid amplification of 5′ complementary DNA (cDNA) ends (5′ RACE) technology used in the protocol does not require primers against *V* genes for targeted enrichment, in contrast to the previous multiplex PCR approach (Extended Data Fig. 2b). They likely represent products of partial or failed recombination and we reasoned that they are still biologically meaningful, reflecting a cell’s history and origin. The Immcantation workflow would divert these contigs into a ‘failed’ file and this file is not typically exposed to the user. Therefore, Dandelion does not automatically filter out nonproductive contigs, and this data have utility, as later discussed.

We also discovered that multiple *J* genes can be sequentially mapped onto different regions in the same mRNA contig, a phenomenon we termed ‘multi-J mapping’. We found that the majority of the most frequent multi-J mapping contigs contained two to four neighboring *J* genes interspersed with introns (Supplementary Table 1). As RNA splicing, rather than DNA recombination, is the process of linking the chosen *J* to *C* genes, contigs with multi-J mapping are likely products of partially spliced transcripts (Fig. 2c). It is biologically plausible that the *J* gene nearest to the 5′ end is the intended exon that would be expressed in the mature mRNA.

We next investigated factors that might contribute to multi-J mapping. We first noted that nonproductive contigs without *V* genes were more likely to have multi-J mapping (Fig. 2c). This difference could be due to nonsense-mediated decay (NMD), an RNA degradation process that is triggered due to premature stop codons^27^. Multi-J mapping contigs containing a *V* gene will initiate translation from the *V* gene, triggering degradation by NMD due to premature stop codons in *J* gene introns, whereas those without a *V* gene cannot be translated and will therefore evade degradation by NMD. To test the contribution of NMD to multi-J mapping, we analyzed peripheral blood mononuclear cells (PBMCs) treated with cycloheximide against control cells. Treatment resulted in an increase in the proportion of multi-J mapping in TCR contigs with *V* genes (Extended Data Fig. 2c), supporting that NMD recognizes and degrades *V*-gene-containing multi-J mapping contigs.

Using a logistic regression model, we found that there was a significant interaction (Benjamini–Hochberg (BH) adjusted *P* = 7.07 × 10^−4^) between *V* gene presence and cycloheximide treatment on multi-J mapping (Fig. 2d; ref. ^3^; Supplementary Table 2, cycloheximide treatment, and Supplementary Table 3), supporting the above findings. However, the significant noninteracting *V* gene term (BH adjusted *P* = 5.73 × 10^−182^) suggests that NMD may only partially account for the effect of *V* genes on multi-J mapping. Furthermore, we found that the known consensus motif for splicing, ‘GTAAGT’ in +1 to +6 position of adjacent intron^28^, was disrupted in *J* genes associated with more multi-J mapping (Fig. 2e and Supplementary Table 4). In conclusion, specific cell types, *J* gene identity, *V* gene presence and NMD are factors that may contribute to multi-J mapping (Extended Data Fig. 2d).

Dandelion also performs γδTCR contig annotation. There are two existing methods for sc-γδTCR mapping as follows: (1) 10X Genomics’ cellranger vdj, (primarily tailored for αβTCR contigs); (2) *TRUST4* (ref. ^29^), which performs de novo contig assembly and annotation. The cellranger software can reconstruct γδTCR contigs but most versions struggle with annotating them (Supplementary Note). While TRUST4 can yield sc-TCR annotations, including γδTCR, it relies on the presence of a *V* gene in the contig thus unable to handle nonproductive contigs without *V* genes. For comparisons, we processed 33 γδTCR libraries^3^; one mapping was done with cellranger 6.1.2 to the 10X GRCh38 5.0.0V(D)J reference, with the contigs identified by cellranger as high confidence subsequently re-annotated with Dandelion. Another mapping was done with cellranger 6.1.2 to the 5.0.0 reference modified to obtain annotated γδTCR contigs as per 10X Genomics’ workaround instructions. We see a consistently higher recovery rate of both high confidence γδTCR contigs and high confidence productive γδTCR contigs in the mapping postprocessed with Dandelion (high confidence contigs: *P* = 5.39 × 10^−7^, high confidence productive contigs: *P* = 3.14 × 10^−6^, Wilcoxon signed-rank test; rank correlations were 1 and 0.98, respectively; Fig. 2f). While 10X Genomics has introduced some γδTCR support with cellranger 7.0.0, the results were inferior to the prior workaround from version 6 (Extended Data Fig. 2e).

### Creating a V(D)J feature space

**Fig. 3:**
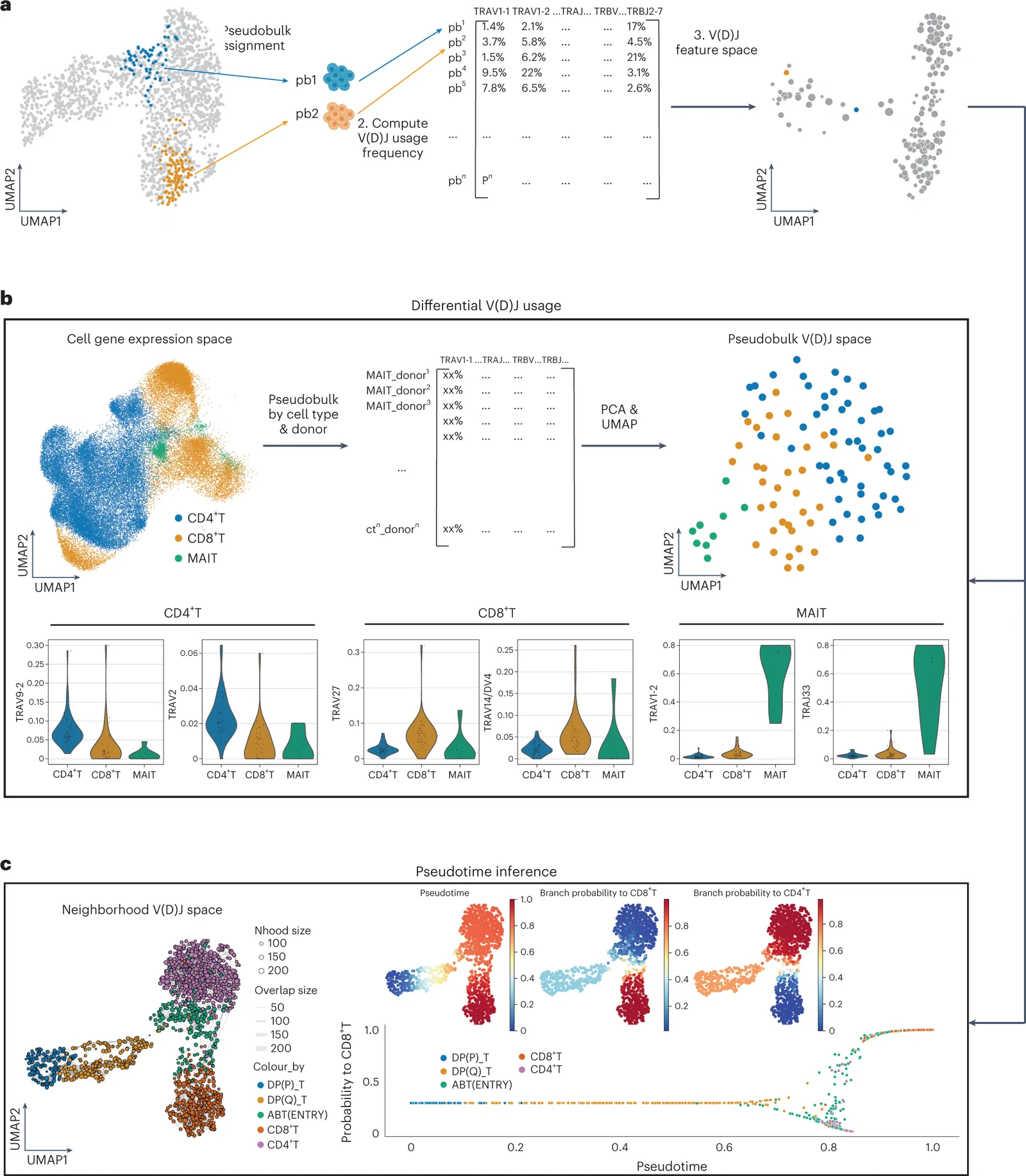
Creating a V(D)J feature space. **a**, Schematic illustration of the workflow of creating a V(D)J feature space. Step 1—cells are assigned to pseudobulks, which can be based on metadata features, or partially overlapping cell neighborhoods. Step 2—V(D)J usage frequency per pseudobulk is computed for each gene and used as input of the V(D)J feature space. Step 3—the V(D)J feature space can be visualized with conventional dimension reduction techniques such as PCA or UMAP, and it can then be used for differential V(D)J usage analysis and pseudotime inference. **b**, Top left—gene expression UMAP of all T cells from adult human tissues in ref. ^5^, colored by low-level cell type annotations. Each point represents a cell. Top middle—V(D)J usage frequency per celltype_donor pseudobulk is computed for each gene and used as input of the V(D)J feature space. Top right—UMAP of the pseudobulk V(D)J feature space of the same cells. Each point represents a cell pseudobulk. Bottom panel—top two differentially expressed TCR genes in CD4^+^ T cells, CD8^+^ T cells and MAIT cells. **c**, Left—UMAP of neighborhood V(D)J feature space covering DP to mature T cells with paired productive αβTCR in data from ref. ^3^. Each point represents a cell neighborhood, colored by the dominant cell type in each neighborhood. The point size represents neighborhood size, with connecting edges representing overlapping cell numbers between any two neighborhoods. Only edges with more than 30 overlapping cells are shown. Right top—inferred pseudotime, and branch probabilities to CD8^+^ T and to CD4^+^ T, respectively, overlaid onto the same UMAP embedding on the left. Right bottom—scatterplot of branch probability to CD8^+^ T against pseudotime. Each point represents a cell neighborhood, colored by the dominant cell type in each neighborhood.

To better leverage the combined gene expression and AIR repertoire data, we introduced an analysis strategy to create a pseudobulked V(D)J feature space, transforming V(D)J data from categorical to continuous format for downstream applications (Fig. 3a). Transcriptionally similar cells are first grouped into pseudo-bulks, which can be based on metadata features, or partially overlapping cell neighborhoods^30^. For instance, cells can be pseudobulked by cell type, donor and organ to perform differential analysis across cell types while controlling for donor and organ differences. For trajectory analysis, we recommend pseudo-bulking cells by partially overlapping cell neighborhoods sampled from gene expression space for example using Milo^30^ to model a more continuous cell state. For each pseudobulk, we compute the fraction of cells using each of the genes in a segment (for example, TRAJ1 to TRAJ61 in the TRAJ segment). The fractions from the various segments are concatenated, forming the V(D)J matrix/space. This can then be used with conventional dimension reduction techniques such as principal component analysis (PCA) or uniform manifold approximation and projection (UMAP).

**Extended Data Fig. 3:**
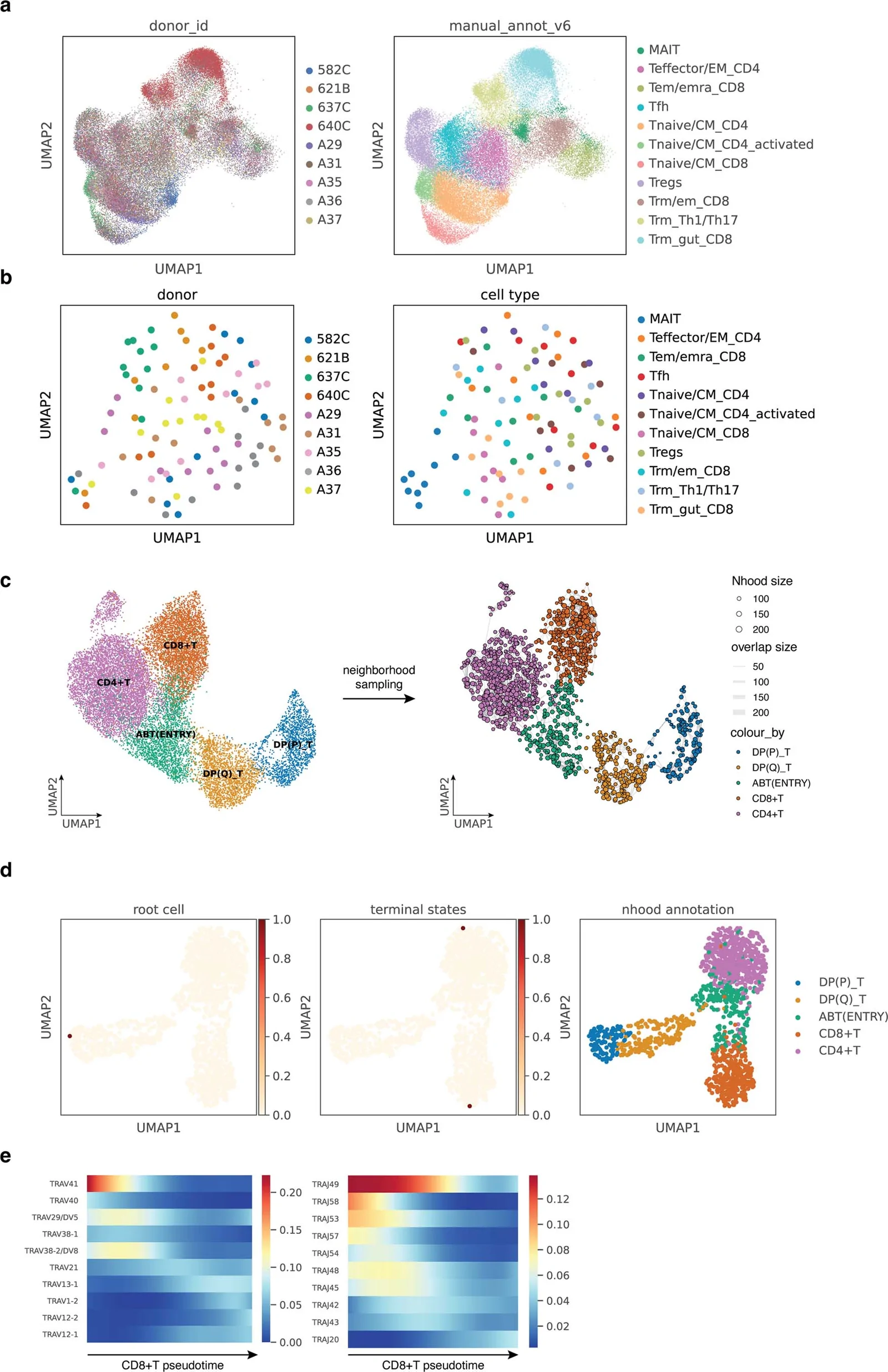
V(D)J feature space. **a**, Gene expression UMAP of all T cells from Conde et al. 2022^5^, colored by donor ID (left) or high-level cell type annotations (right). Each point represents a cell. **b**, UMAP of the pseudo-bulk V(D)J feature space of the same cells as in **a**, colored by donor ID (left) or high-level cell type annotations (right). Each point represents a cell pseudo-bulk. **c**, Left: UMAP of DP to mature T cells with paired productive αβTCR in data from Suo et al. 2022^3^. Each point represents a cell, colored by cell types. Right: cell neighborhood graph on the same UMAP embedding. Each point represents a cell neighborhood, colored by cell types. The point size represents neighborhood size, with connecting edges representing overlapping cell numbers between any two neighborhoods. Only edges with more than 30 overlapping cells are shown. The layout of nodes is determined by the position of the neighborhood index cell in the UMAP on the left. **d**, The root cell and terminal states selected for pseudotime inference in Fig. 3c. **e**, Gene expression trends over CD8 + T pseudotime imputed with *Palantir*^32^. Only the top 10 most frequently used TRAV or TRAJ genes are shown.

One utility of this V(D)J space is demonstrated as we pseudobulked adult human T cells^5^ by cell types and donors to explore differential usage that is consistent across different donors. On the V(D)J feature space-based UMAP, pseudobulks containing mucosal-associated invariant T (MAIT) cells formed a distinct cluster, in contrast to the single-cell gene expression-based UMAP (Fig. 3b and Extended Data Fig. 3a,b). This is expected due to the semi-invariant nature of MAIT TCRs and illustrates the power of the V(D)J feature space. Although there is no clear clustering in other cell types apart from MAIT (Extended Data Fig. 3b), CD4^+^ T cells were distinctly separated from CD8^+^ T cells (Fig. 3b). Differential V(D)J usage for each cell type can be computed, for example, with nonparametric statistical tests implemented within scanpy^14^ (Fig. 3b and Supplementary Table 5).

### Leveraging V(D)J usage in pseudotime trajectory inference

We also developed a new usage for V(D)J data by performing pseudotime inference on the cell neighborhood-based V(D)J feature space. Many pseudotime inference methods have been proposed based on transcriptome similarity^31^. However, current approaches remain problematic in immune cell development because the differentiation process is often interspersed with waves of proliferation, and transcriptomic convergence, for example, between NKT and NK cells can be misleading. Because the usage of *V*(*D*)*J* genes in AIRs changes definitively as a result of cycles of recombination and selection during lymphocyte development, the AIR repertoire acts as a natural ‘time-keeper’ for developing T and B cells. A developing T cell’s fate toward CD8 versus CD4 T cells is determined by whether its TCR interacts with antigen presented on MHC class I or class II during positive selection. Therefore, it is biologically conceivable that the TCR gives more accurate predictions on the branch probability of each T cell lineage. For this task, we chose to pseudobulk by cell neighborhoods as modeling cell states with partially overlapping cell neighborhoods has advantages over clustering into discrete groups.

**Fig. 4:**
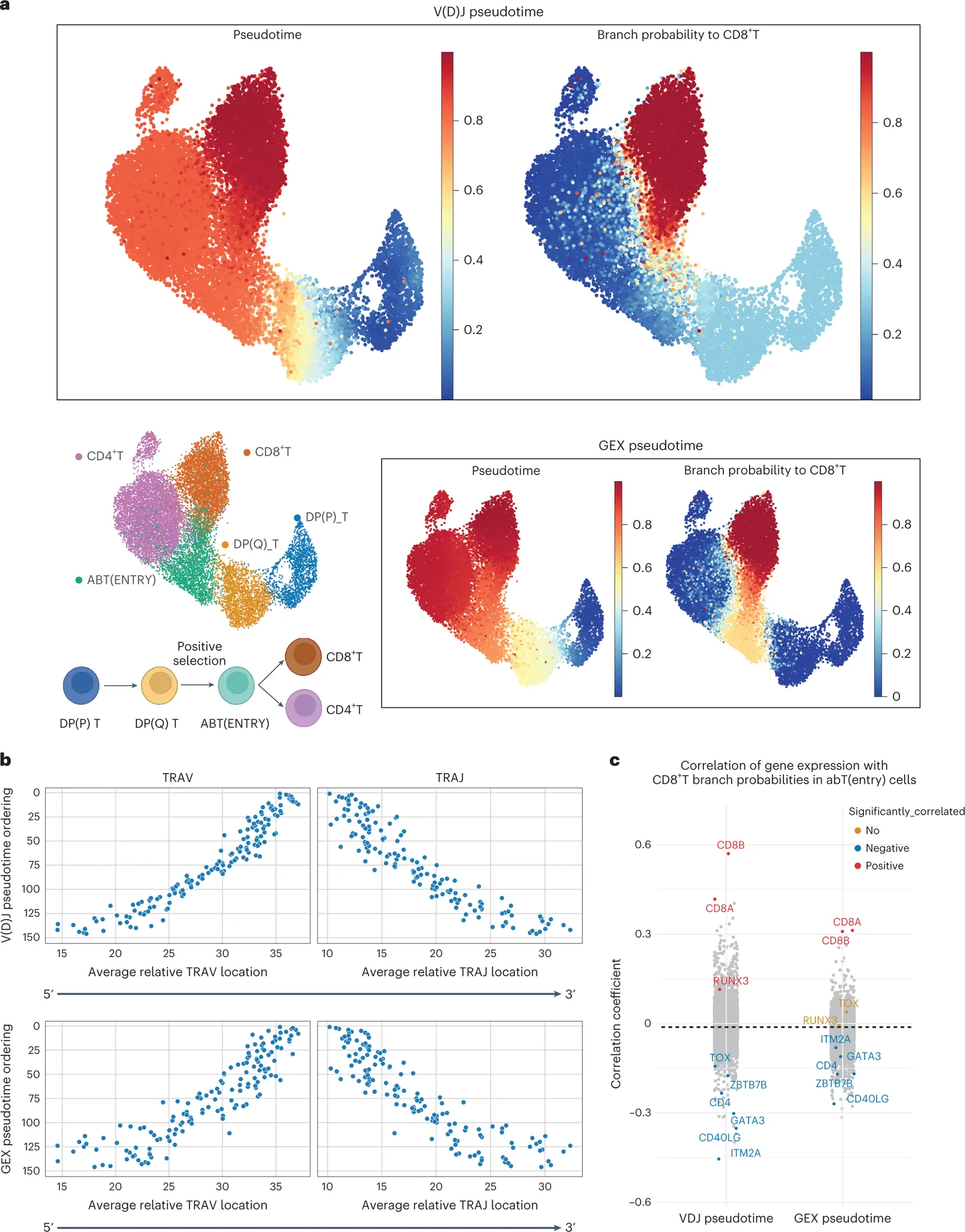
Comparing pseudotime inferred from V(D)J space or gene expression (GEX) space. **a**, Top—pseudotime and branch probability to CD8^+^ T inferred from neighborhood V(D)J space in Fig. 3c, projected back to the cells, overlaid onto the same UMAP embedding as in the top left panel. Left bottom—UMAP of DP to mature T cells with paired productive αβTCR in data from ref. ^3^. Each point represents a cell, colored by cell types. Underneath the UMAP is a schematic showing the T cell differentiation process. Right bottom—pseudotime and branch probability to CD8^+^ T inferred from neighborhood GEX space, projected back to the cells, overlaid onto the same UMAP embedding as in the top left panel. **b**, Scatterplots of the pseudotime ordering against the average relative TRAV or TRAJ location. Each point represents a cell neighborhood. Each *TRAV* or *TRAJ* gene is encoded numerically for its relative genomic order. The *x* axis represents the average TRAV/TRAJ relative location for each cell neighborhood. Top—results from pseudotime inferred from neighborhood V(D)J space. Bottom—results from pseudotime inferred from neighborhood GEX space. **c**, Stripplot of correlation coefficients of gene expression with branch probabilities to CD8^+^ T within abT(entry) cells, for branch probabilities inferred from neighborhood V(D)J space and neighborhood GEX space separately. Only genes that are known CD4^+^/CD8^+^ T cell markers or TFs involved in CD8^+^ T/CD4^+^ T lineage decisions are labeled and colored. The rest of the genes are grayed out. Labeled genes that had significant (BH adjusted *P* < 0.05) positive correlations were colored in red, the ones with significant negative correlations were colored in blue and those without significant correlations were colored in orange.

We sampled cell neighborhoods from developing T cells with productive αβTCR (double positive (DP) to mature single positive CD4^+^/CD8^+^ T cells)^3^ on a k-nearest neighbor graph built with gene expression data using Milo^30^ and constructed the neighborhood V(D)J feature space (Fig. 3c and Extended Data Fig. 3c). Trajectory analysis on this V(D)J feature space was performed using Palantir^32^. It outputs pseudotime and branch probabilities (Fig. 3c) to each terminal state (Extended Data Fig. 3d). The inferred pseudotime follows from proliferating to quiescent DP ((DP(P)) and DP(Q)) T cells, to abT(entry), which splits into CD8^+^ T and CD4^+^ T lineages. TCR usage trend can be visualized along the pseudotime trajectory (Extended Data Fig. 3e). The average pseudotime and branch probabilities per cell can also then be projected back from its neighborhoods (Fig. 4a).

**Extended Data Fig. 4:**
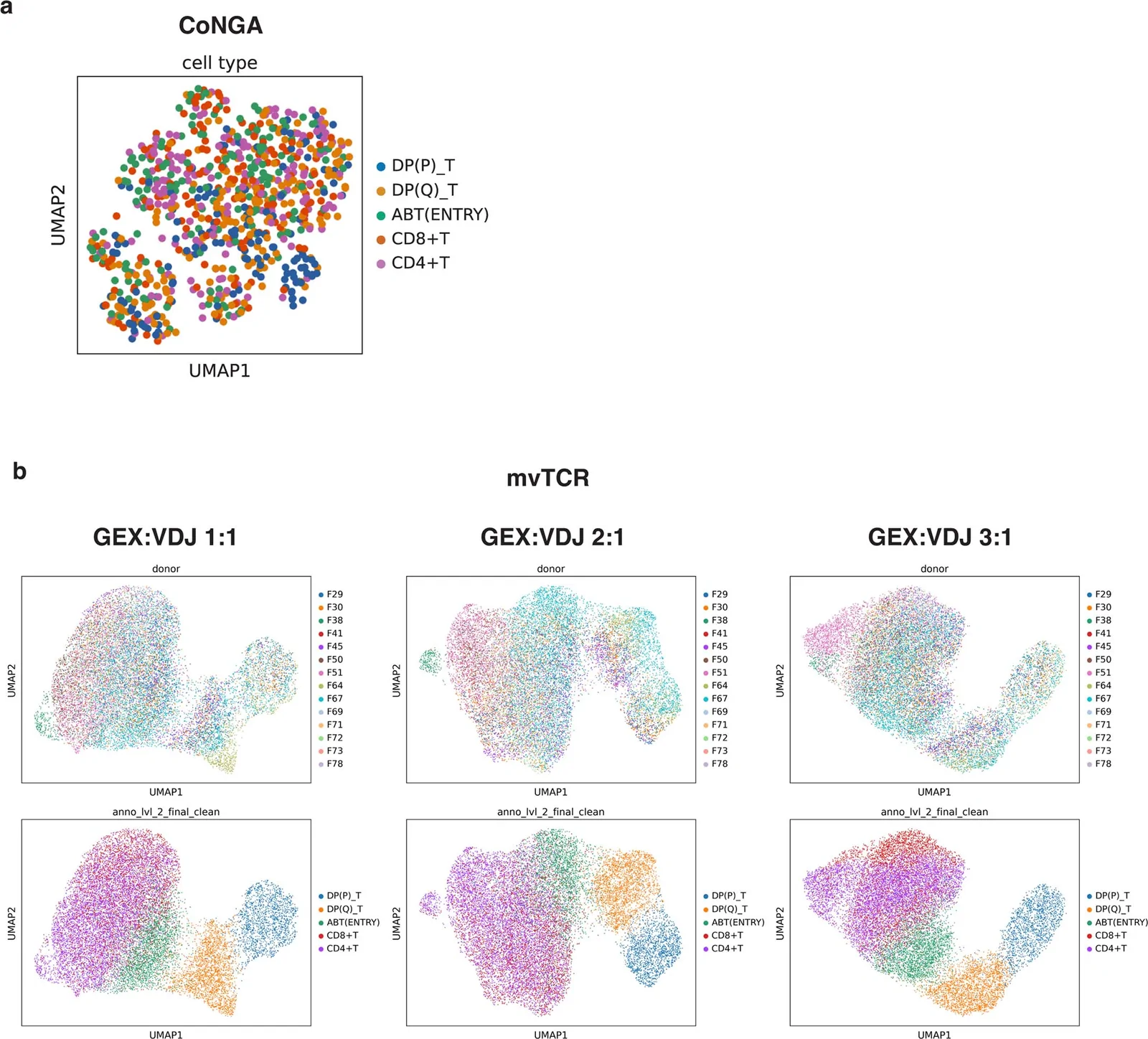
Embedding with alternative methods. **a**, UMAP representation of tcrdist-derived PCA coordinates of VDJ data computed by CoNGA^21^, with the same dataset as used in Supplementary Fig. 3c, colored by cell types. **b**, UMAP representation of joint gene expression and TCR embedding computed by mvTCR^22^ with varying weights for GEX and VDJ input, on the same dataset as used in Supplementary Fig. 3c. Cells are colored by donor ID (top panel) or cell types (bottom panel).

There are two alternative tools, CoNGA^21^ and mvTCR^22^, that integrate transcriptome with TCR information. Both were created to detect clonally expanded cell types with CDR3 sequences being the input. We tested whether they could also be used to reveal developmental relationships with the same dataset above. Both CoNGA and mvTCR failed to preserve the intercellular relationships (Extended Data Fig. 4a,b). This is not surprising, as selection of different *V*(*D*)*J* genes underpins recombination, while CDR3 diversity can additionally be influenced by random nucleotide insertions.

### V(D)J trajectory accurately orders T cell development

We next compared the results of trajectory inference on feature spaces from pseudobulked neighborhood V(D)J, pseudobulked neighborhood gene expression or single-cell gene expression.

**Extended Data Fig. 5:**
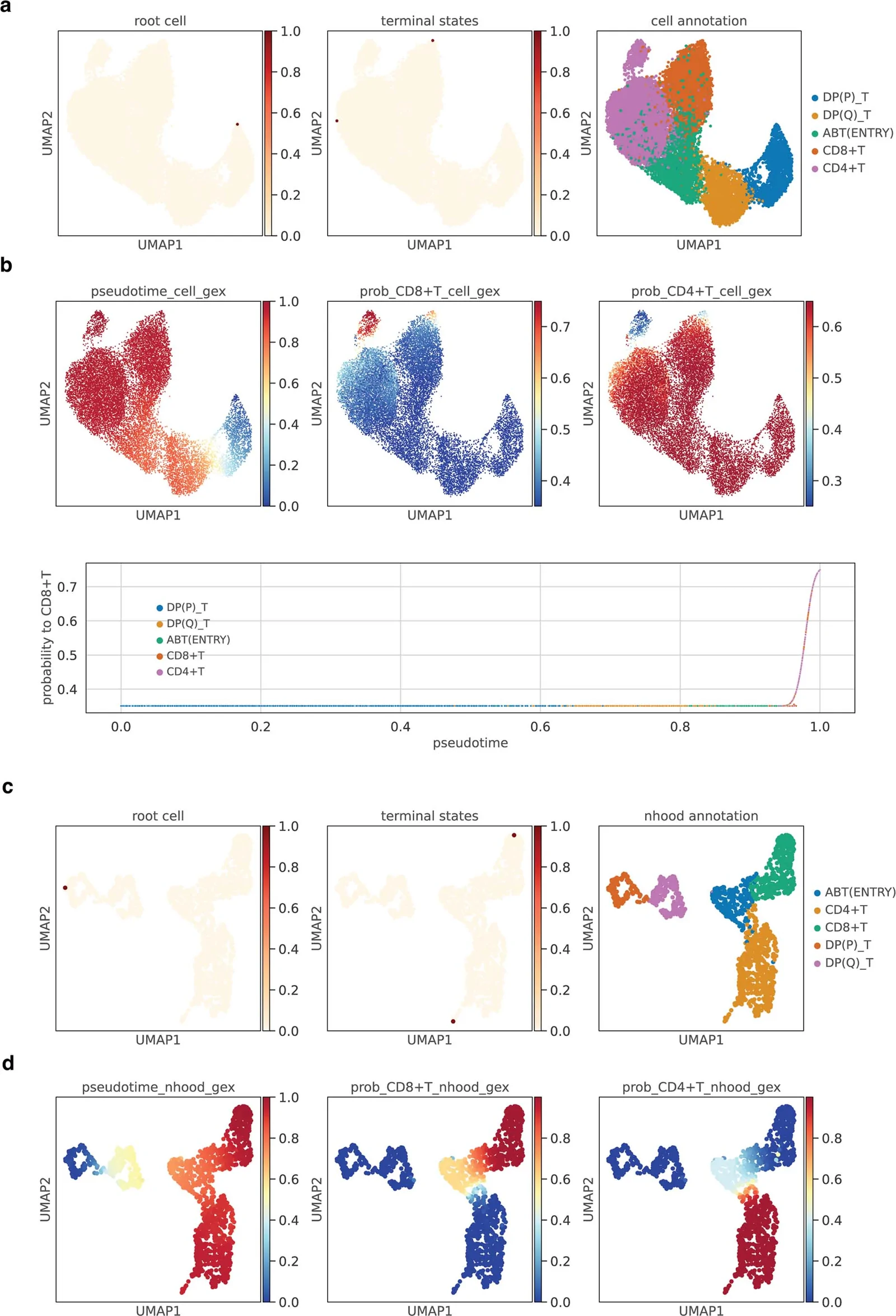
T cell development pseudotime inference comparison. **a**, DP to mature T cells with paired productive αβTCR in data from Suo et al. 2022^3^, on the same UMAP embedding as in Fig. 4a and Supplementary Fig. 3c. The first two panels show the root cell and terminal states selected for pseudotime inferred directly from single-cell gene expression. The last panel shows the cell types. **b**, Top: pseudotime and branch probabilities inferred directly from single-cell gene expression on the same UMAP embedding as in **a**. Bottom: scatterplot of branch probability to CD8+ T against pseudotime. Each point represents a cell. **c**, UMAP of neighborhood GEX space, with the same neighborhoods as sampled in Supplementary Fig. 3c and UMAP embedding computed on gene expression pseudo-bulked by neighborhoods. Each point represents a cell neighborhood. The first two panels show the root cell and terminal states selected for pseudotime inferred from neighborhood GEX space. The last panel shows the cell types. **d**, Inferred pseudotime, and branch probabilities to CD8+ T and to CD4+ T respectively overlaid onto the same UMAP embedding in **c**.

The analysis of single-cell gene expression performed unsatisfactorily as a large proportion of CD8^+^ T and CD4^+^ T cells were misclassified with higher branch probabilities to the opposite terminal state (Extended Data Fig. 5a,b). Therefore, we mainly focused our comparison with results from pseudobulked neighborhood gene expression (GEX) space, which produced more biologically meaningful pseudotime and branch probabilities (Fig. 4a). When projected back to cells, the inferred pseudotime in the pseudobulked space better reflected the known biology of DP(P)_T to DP(Q)_T, to abT(entry) and subsequent splits into CD8^+^ T and CD4^+^ T lineages (Extended Data Fig. 5c,d). This suggests that pseudotime inference with pseudobulked cells works better than directly from single cells, potentially due to less noise compared to single-cell data.

**Extended Data Fig. 6:**
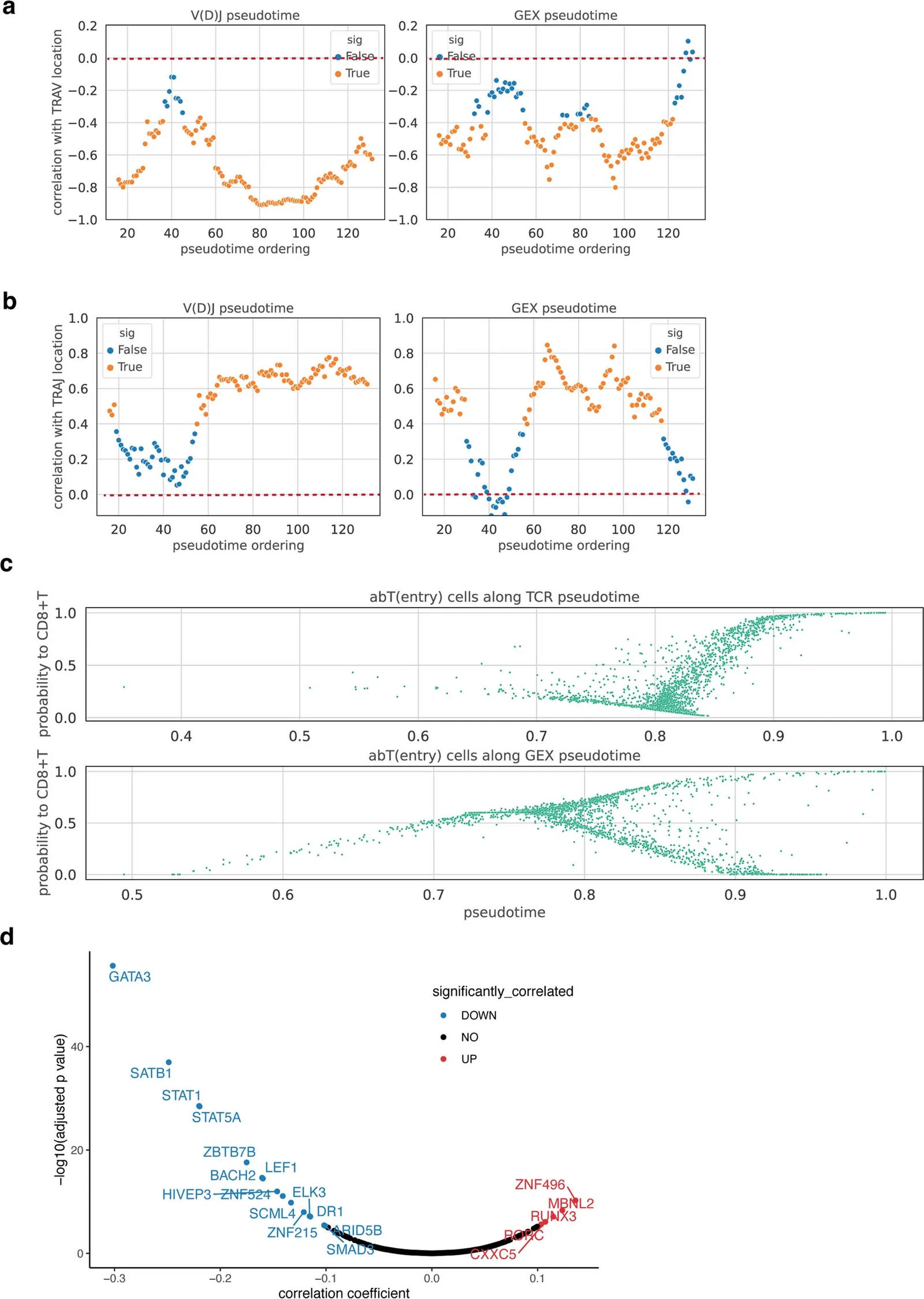
Comparing pseudotime inferred from neighborhood V(D)J space or GEX space. **a**, Pearson’s correlation coefficients of pseudotime order and average relative TRAV location over sliding windows of 30 adjacent neighborhoods on the pseudotime order (left: pseudotime inferred from neighborhood V(D)J space; right: pseudotime inferred from neighborhood GEX space). *Y*-axis is the correlation coefficient and the x-axis is the median pseudotime order of the 30 adjacent neighborhoods. The color of the points represents statistical significance (orange: *P*-value from the Pearson’s correlation < 0.05; blue: *P*-value ≥ 0.05). The red dashed lines mark the correlation coefficient of 0. **b**, The same plot as in a but for TRAJ. **c**, Scatterplots of branch probability to CD8+ T against pseudotime in abT(entry) cells. Each point represents a cell. Top panel: pseudotime inferred from neighborhood V(D)J space as in Fig. 4a top panel. Bottom panel: pseudotime inferred from neighborhood GEX space as in Fig. 4a bottom right panel. **d**, Volcano plot summarizing results of TFs that are correlated with branch probabilities to CD8+ T lineage in V(D)J pseudotime within abT(entry) cells. The *y*-axis is the -log_10_(BH adjusted *P*-value) and the *x*-axis is the correlation coefficient. Labeled TFs that had significant (BH adjusted *P*-value < 0.05) positive correlations (correlation coefficient > 0.1) were colored in red, the ones with significant negative correlations (correlation coefficient < −0.1) were colored in blue, and the rest were colored in black.

We observed two major differences when comparing the pseudotime inferred from neighborhood V(D)J feature space versus that from neighborhood GEX space (Fig. 4a). First, the DP(Q) T cells dwelled for a longer ‘time’ in the V(D)J trajectory compared to the GEX trajectory. Second, the branching point of CD8^+^ T and CD4^+^ T cell lineages happened earlier in abT(entry) cells in the V(D)J trajectory (Extended Data Fig. 6c). To assess the fidelity of the V(D)J trajectory, we used the known fact that V–J recombination in the TRA locus happens processively^33^ (from middle to distal ends on the genome). We encoded the genomic order numerically for each *TRAV* and *TRAJ* gene and the V(D)J pseudotime ordering for each DP(Q) neighborhood showed a substantially better monotonic relationship with the TRAV relative locations (Fig. 4b). Average local correlations across adjacent neighborhoods along V(D)J pseudotime had higher absolute correlation coefficients on average (−0.67 versus −0.43 for TRAV; Extended Data Fig. 6a). A smaller improvement was also observed for *TRAJ*, with the average local Pearson’s correlations improved from 0.42 to 0.50 (Extended Data Fig. 6b).

CD4 versus CD8 T cell lineage commitment is a classical immunological binary lineage decision that has been intensely investigated over many years^34^ but remains challenging to study as the selection intermediates have been difficult to observe directly^35^. We examined which genes in abT(entry) cells showed expression patterns that are correlated with branch probabilities to CD8^+^ T versus CD4^+^ T lineage (Fig. 4c).

The top genes that were positively correlated with the CD8^+^ T cell lineage choice included *CD8A* and *CD8B*, which are markers for CD8^+^ T cells^6^. The top genes that were negatively correlated included *CD40LG*, which is a marker for CD4^+^ T helper cells^6^, and *ITM2A* (induced during positive selection and causes CD8 downregulation^36^). Other markers of CD4^+^ T cells such as *CD4* (ref. ^6^), together with highly validated transcription factors (TFs) that are known to be involved in CD8^+^ T or CD4^+^ T lineage decisions^34^, including *RUNX3* (ref. ^37,38^), *ZBTB7B*^39,40^, *TOX*^41^ and *GATA3* (ref. ^42,43^), all displayed significant correlations in the expected directions. However, when using GEX pseudotime, the correlations were notably reduced and some (for example, *TOX* and *RUNX3*) were no longer statistically significant (Fig. 4c). For *TOX*, the direction of the correlation was wrongly inverted (Fig. 4c). In addition, the V(D)J pseudotime also revealed new associations for TFs such as *ZNF496*, *MBNL2* and *RORC* for CD8^+^ T, and *SATB1, STAT5A* and *STAT1* for CD4^+^ T (Extended Data Fig. 6d and Supplementary Table 6).

**Extended Data Fig. 7:**
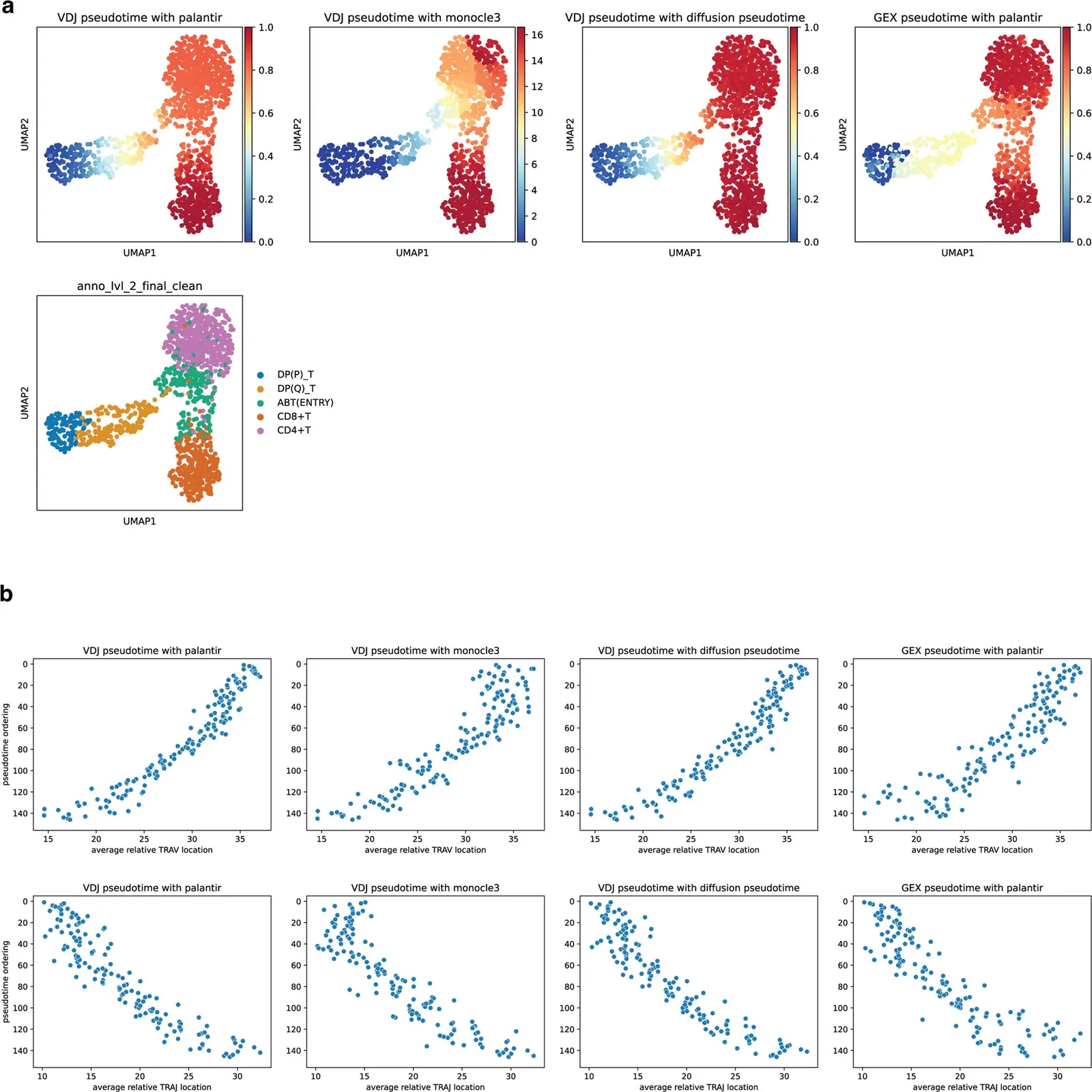
Pseudotime inferred with different trajectory inference methods. **a**, First three panels display pseudotime inferred from neighborhood V(D)J space using *Palantir*^32^, *monocle3*^44^, and *diffusion pseudotime*^45^ respectively, overlaid onto the same UMAP embedding as in Fig. 3c with each point represents a cell neighborhood. The fourth panel represents the pseudotime inferred from neighborhood GEX space using *Palantir*^32^. The last panel represents the dominant cell type in each neighborhood. b, Scatterplots of the pseudotime ordering against the average relative TRAV (top) and TRAJ (bottom) location. Each point represents a cell neighborhood. Each TRAV or TRAJ gene is encoded numerically for its relative genomic order. The *x*-axis represents the average TRAV/TRAJ relative location for each cell neighborhood. The *y*-axis represents the pseudotime order inferred from neighborhood V(D)J space using *Palantir*^32^, *monocle3*^44^, and *diffusion pseudotime*^45^, and the pseudotime order inferred from neighborhood GEX space using *Palantir*^32^ respectively. The Pearson’s correlations are −0.95, −0.91, −0.95, and −0.90 respectively (*P*-values of 4.8e-76, 4.9e-56, 2.1e-74, and 7.4e-54) for TRAV, and 0.93, 0.90, 0.93, and 0.89 respectively (*P*-values of 1.7e-62, 3.8e-54, 7.6e-65, and 4.2e-52) for TRAJ.

We have also used different pseudotime inference methods to ensure the robustness of the results. Neighborhood V(D)J-based pseudotime trajectories inferred using monocle3 (ref. ^44^) and diffusion pseudotime^45^ similarly showed a better monotonic relationship with TRAV/TRAJ relative locations compared to neighborhood GEX-based pseudotime (Extended Data Fig. 7a,b). Overall, Palantir is preferred as it outputs the branch probabilities, which is useful in deciphering CD4/8 lineage decisions.

Taken together, we showed that V(D)J-based pseudotime inference gives more accurate DP(Q) T cell alignment and improves lineage association within abT(entry) cells. We can use this approach to recapitulate known regulators and uncover new candidate regulators underlying CD8^+^ T/CD4^+^ T fate choice.

### Using nonproductive recombination as a ‘fossil record’

**Fig. 5:**
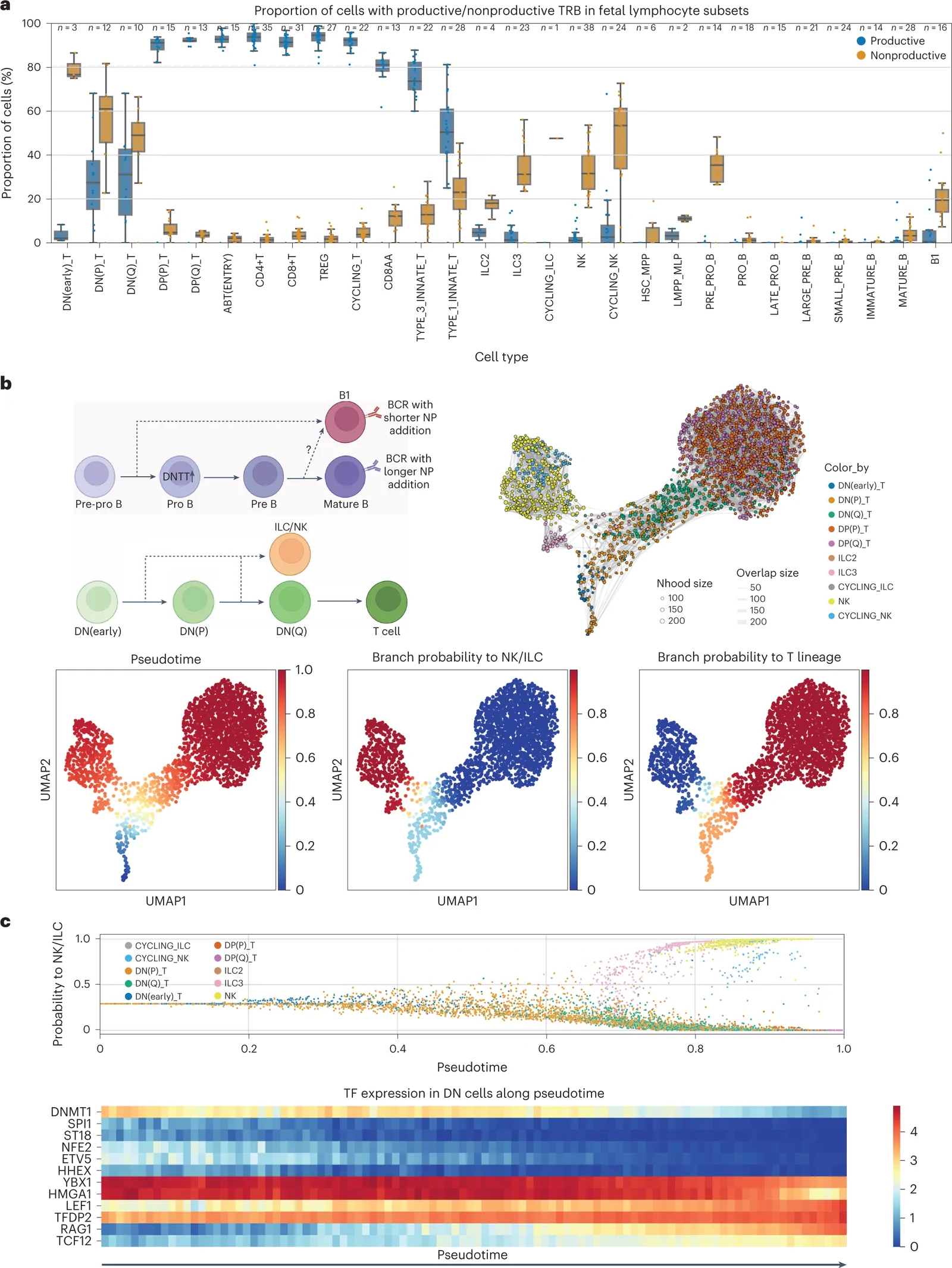
Insights into lymphocyte development from nonproductive TCR. **a**, Boxplot of the proportion of cells with productive (blue) or nonproductive (orange) TRB in different fetal lymphocyte subsets. Each point represents a sample and data were taken from ref. ^3^. Only samples with at least 20 cells are shown. Boxes capture the first to third quartiles and whisks span a further 1.5× interquartile range on each side of the box. The annotations used here were based on the version whereby the exact identity of cycling B cells was predicted to be immature B, mature B, B1 or plasma B cells using Celltypist^3,5^. The equivalent boxplot using the original annotations is shown in Extended Data Fig. 8a. **b**, Top left—schematic illustration showing the proposed development of B cells (top panel), and the relationship between ILC/NK and T cell lineages. Top right—UMAP of neighborhood V(D)J feature space covering ILC, NK and developing T cells with TRBJ in data from ref. ^3^. Each point represents a cell neighborhood, colored by cell types. The point size represents neighborhood size, with connecting edges representing overlapping cell numbers between any two neighborhoods. Only edges with more than 30 overlapping cells are shown. Bottom—inferred pseudotime and branch probabilities to ILC/NK and T lineage, respectively, overlaid onto the same UMAP embedding on the top right. **c**, Top—scatterplot of branch probability to ILC/NK lineage against pseudotime. The pseudotime was inferred from neighborhood V(D)J space shown in Fig. 5b and projected back cells. Each point represents a cell, colored by cell types. Bottom—heatmap of TF expressions across pseudotime in DN T cells. Pseudotime is equally divided into 100 bins, and the average gene expression is calculated for DN T cells with pseudotime that falls within each bin. Genes selected here are TFs that had significantly high Chatterjee’s correlation^73^ with pseudotime (BH adjusted *P* < 0.05, and correlation coefficient >0.1).

**Extended Data Fig. 8:**
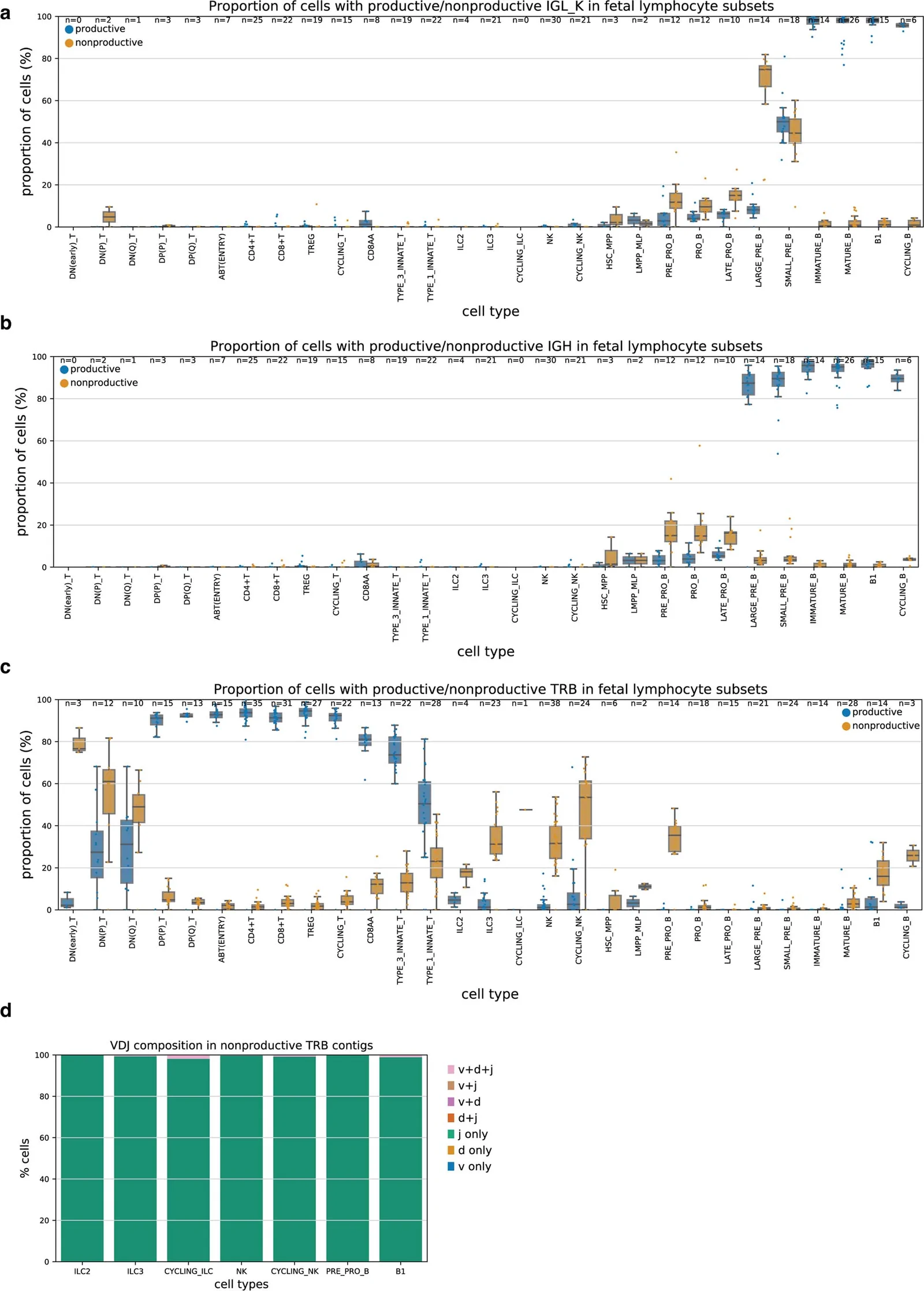
Non-productive BCR and TCR. **a,b,c**, Boxplot of the proportion of cells with productive (blue) or non-productive (orange) BCR light chain (**a**) and heavy chain (**b**), and TRB (**c**) in different fetal lymphocyte subsets. Each point represents a sample and data were taken from Suo et al. 2022^3^. Only samples with at least 20 cells are shown. Boxes capture the first to third quartiles and whisks span a further 1.5X interquartile range on each side of the box. **d**, Barplot showing the VDJ composition of non-productive TRB contigs in selected lymphocyte subsets from Fig. 5a.

Based on our earlier observations of high proportions of nonproductive contigs being represented in the single-cell V(D)J data (Fig. 2a), we next explored whether this was cell-type specific. As expected, nonproductive BCR contigs were restricted to B lineage cells (Extended Data Fig. 8a,b). However, nonproductive TRB contigs were surprisingly expressed in many cell-type lineages (developing DN T cells, ILC/NK, B cells; Fig. 5a and Extended Data Fig. 8c). The majority of the nonproductive TRB contigs within ILC/NK/B cells were contigs without *V* gene (Extended Data Fig. 8d).

**Extended Data Fig. 9:**
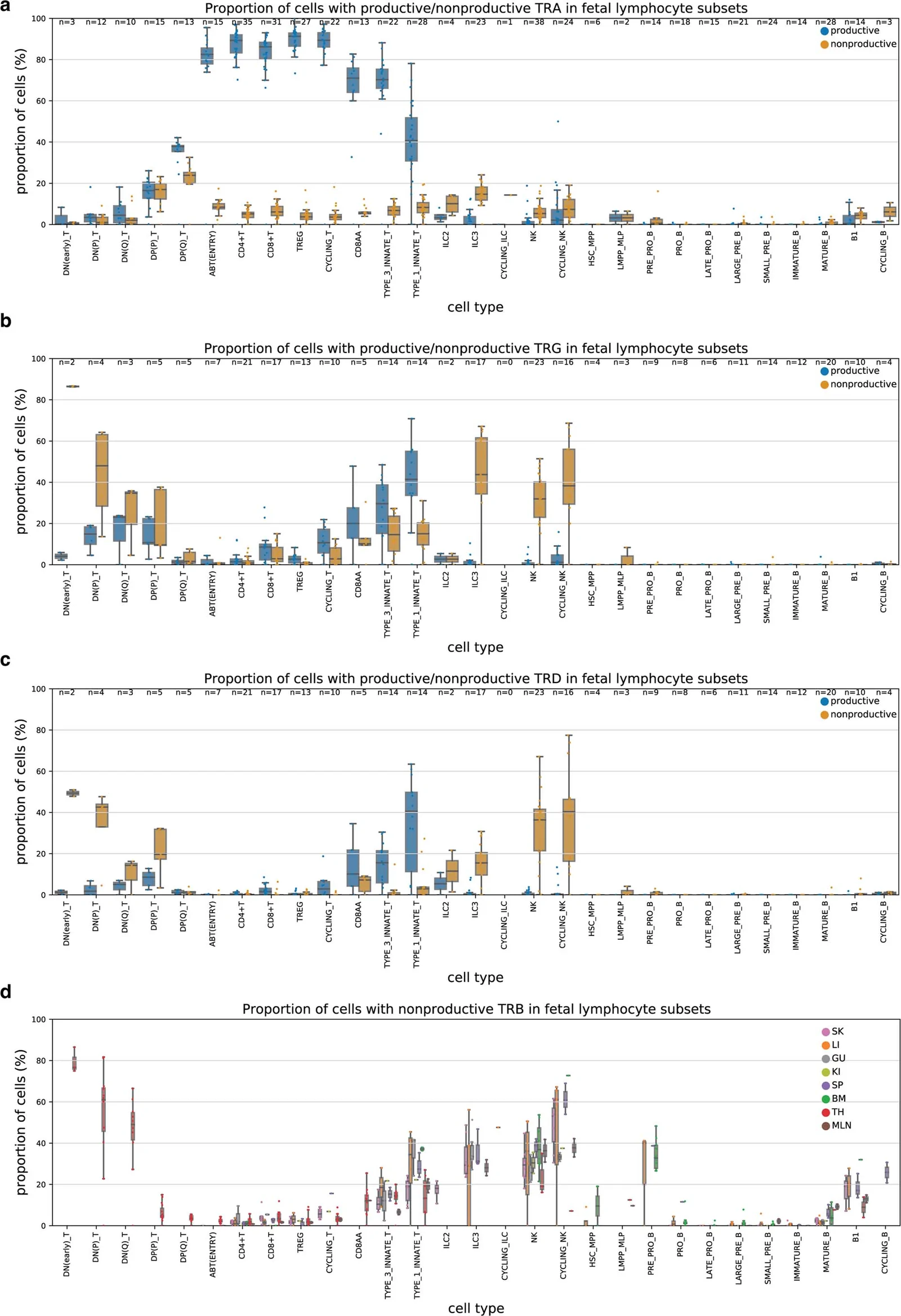
Non-productive TCR. **a**–**c**, Boxplot of the proportion of cells with productive (blue) or non-productive (orange) TRA (**a**), TRG (**b**) and TRD (**c**) in different fetal lymphocyte subsets. Each point represents a sample and data were taken from Suo et al. 2022^3^. Only samples with at least 20 cells are shown. Boxes capture the first to third quartiles and whisks span a further 1.5X interquartile range on each side of the box. **d**, Boxplot of the proportion of cells with non-productive TRB in different fetal lymphocyte subsets, colored by organs. Each point represents a sample. Only samples with at least 20 cells are shown. Boxes capture the first to third quartiles and whisks span a further 1.5X interquartile range on each side of the box. Sample sizes (n) of all box plots from left to right are 3, 12, 10, 15, 13, 13, 1, 1, 4, 2, 12, 3, 2, 8, 3, 1, 2, 1, 12, 3, 2, 8, 2, 1, 2, 12, 3, 2, 7, 1, 12, 1, 2, 6, 1, 12, 1, 2, 8, 2, 2, 4, 3, 1, 4, 2, 7, 3, 2, 6, 3, 1, 4, 5, 3, 2, 7, 6, 1, 7, 8, 3, 3, 2, 8, 5, 2, 7, 1, 1, 3, 2, 7, 2, 1, 4, 2, 1, 1, 3, 10, 1, 4, 11, 1, 2, 3, 10, 1, 1, 7, 10, 1, 2, 1, 7, 9, 2, 5, 1, 4, 3, 1, 6, 5, 6, 1, 3, 2, 8, 2, 1, 3, 1, 2, 3, 2, 3, 3.

The B lineage cells with nonproductive TRB contigs included pre–pro B and B1 cells but not pro- or pre-B cells (Fig. 5a and Extended Data Fig. 8c). Pre–pro B and B1 cells expressed only nonproductive TRB but not TRG/D contigs (Extended Data Fig. 9a–c), suggesting that they share a common developmental route (Fig. 5b), bypassing pro-/pre-B cell stages. This clarifies that human fetal B1 cells can emerge through an alternative route. The conventional route is thought to progress from pre–pro, pro-, pre-, immature, to mature naive B cells^46^. Our observations are consistent with findings in mouse B1s, which were shown to bypass the pre-BCR selection stage^47,48^, which normally happens in pre-B cells to remove self-reactive B cells. This may also explain why B1 cells have BCRs with shorter noncoded/palindromic (N/P) nucleotide insertions^3^, due to negligible expression of DNTT in pre–pro B but much higher expression in pro- and late pro-B cells^3^. In addition, as pre–pro B cells are almost undetectable in adult bone marrow^49^, it potentially explains the difficulty of identifying B1-like cells in adult human tissues^3^.

**Extended Data Fig. 10:**
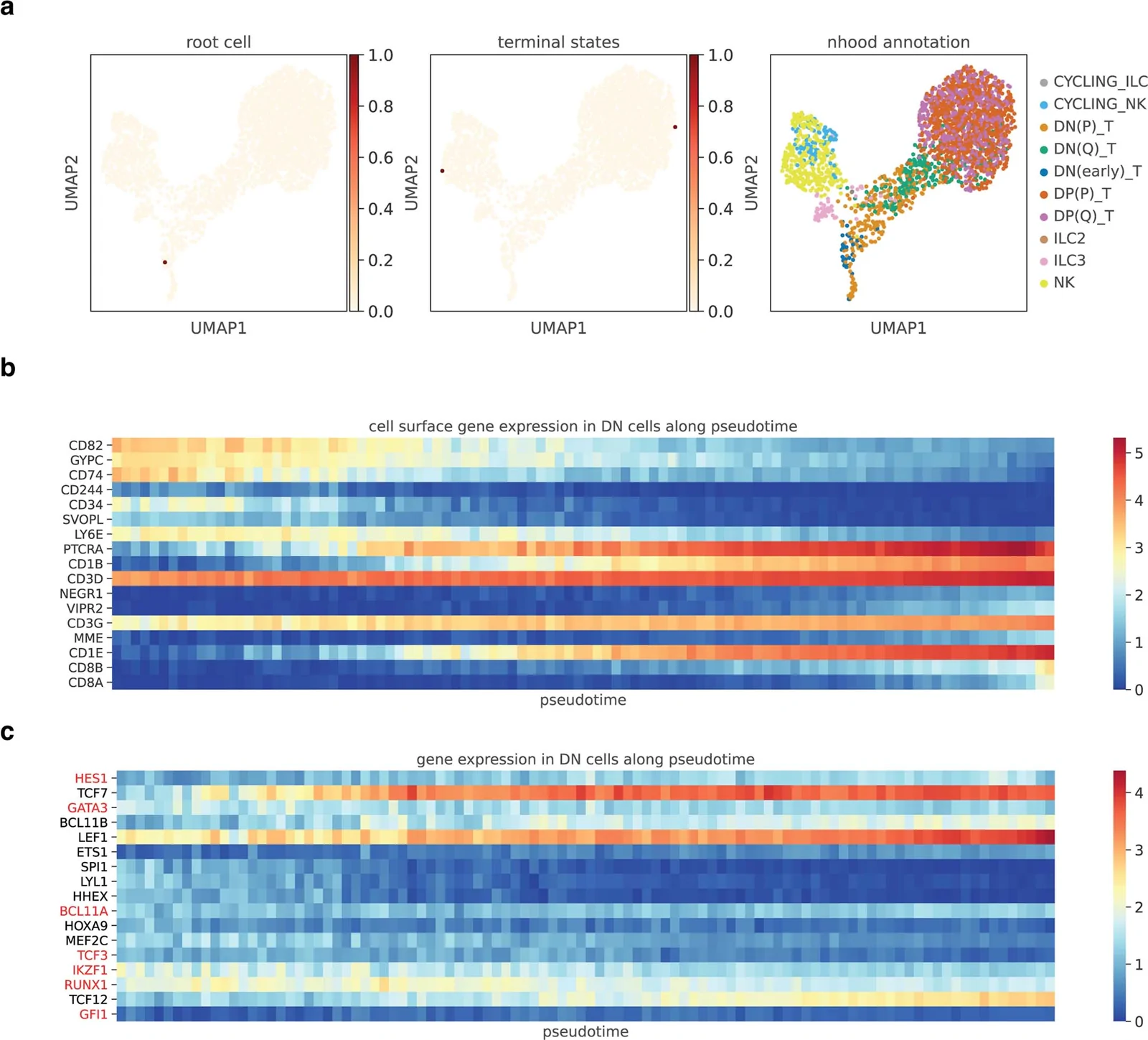
TRBJ-based trajectory for ILC/NK/T cell lineage. **a**, Neighborhood V(D)J feature space covering ILC, NK and developing T cells with TRBJ on the same UMAP embedding as in Fig. 5b. The first two panels show the root cell and terminal states selected for pseudotime inference. The last panel shows the cell types. **b**, Heatmap of gene expression for genes encoding cell surface proteins across pseudotime in DN T cells. Pseudotime is equally divided into 100 bins, and the average gene expression is calculated for DN T cells with pseudotime that falls within each bin. Genes selected here had significantly high Chatterjee’s correlation with pseudotime (BH adjusted *P*-value < 0.05, and correlation coefficient > 0.1). **c**, Heatmap of gene expression for TFs known to be important in mouse DN T cell development^53^, across pseudotime in human fetal DN T cells. TFs that showed discordant expression patterns between mouse and human are highlighted in red.

Similar to DN T cells, the ILC/NK lineage also expressed nonproductive TRG/D contigs with some TRA contigs (Extended Data Fig. 9a–c). We used TRBJ frequency to construct a V(D)J feature space because all T/ILC/NK cells express TRBJ (Fig. 5b and Extended Data Fig. 10a). The inferred trajectory suggests that ILC/NK cells deviate away from T cell development between DN(early) and DN(Q) stage (Fig. 5b,c).

Previous literature on the ILC/NK lineage has also demonstrated partial recombination of TRG/D in mouse lung ILC2 (ref. ^50^) and of TRB/G in mouse thymic ILC2 (ref. ^51^), leading to the hypothesis of ‘aborted’ DNs for ILC/NK development^52^. Our observation of the expression of nonproductive TRB/G/D in ILC/NK cells partially supports this theory. Notably, we also observed nonproductive TRB expression in ILC/NK cells in other fetal organs, with no overt differences in frequencies between organs (Extended Data Fig. 9d). This potentially suggests that T cells and ILC/NK cells might share the same initial stage of development, and then deviate away from each other before productive TRB/TRG/TRD is made.

We observed that expression levels of genes encoding TFs (Fig. 5c) and cell surface proteins (Extended Data Fig. 10b) such as *SPI1*, *RAG1*, *HHEX*, *TCF12*, *CD34*, *CD3D*, *CD8A* and *CD8B*, followed an expected pattern along the TRBJ-inferred trajectory^53^. At the same time, we also discovered many new genes that could redefine DN stages at a higher resolution than previously reported in the literature. We note that there were some discordances in expression patterns of selected TFs between human and mouse DN development^53^ (Extended Data Fig. 10c). However, this discrepancy could be due to age mismatch that is fetal human to adult mouse, and the mouse data was mainly learned from TF-knockout studies. Future work with detailed comparison using paired scRNA-seq and scVDJ-seq in mice and humans of different age groups will help provide clarity to this.

Finally, we repeated the analysis in human fetal myeloid cells^3^ to ask whether the pDCs that have initiated BCR rearrangements are derived from lymphoid progenitors^54,55^. pDC can be derived from both myeloid and lymphoid lineages^54,55^ and there is IgH D-J rearrangement in some pDCs^54,56–59^. We found some nonproductive BCR in pDC (both heavy and light chain as shown in Supplementary Fig. 1a,b) in agreement with previously reported IgH D-J rearrangement in pDC^54,56–59^. However, pDC itself expresses RAG and DNTT (Supplementary Fig. 1c). The presence of nonproductive BCR does not necessarily indicate that pDCs are derived from lymphoid progenitors as BCR rearrangement can be carried by RAG in pDC itself^57^. While it may be interesting to use our VDJ-based trajectory to explore whether the development of pDC overlaps with early B cell development, the current dataset is limited by the cell number as only 51 pDC and cycling pDC cells have nonproductive IGH.

In summary, the unexpected finding of expression of nonproductive TCR contigs in specific cell types has the potential to shed new light on lymphocyte development. Our analysis suggests that B1 potentially arises directly from pre–pro B cells and provides support for the ‘aborted’ DN theory for ILC/NK cell origins.

## Discussion

Overall, Dandelion improves upon existing methods with more refined contig annotations, recognizing nonproductive contigs, identifying multi-J mapping and recovering more γδTCR contigs. Pseudotime inference on V(D)J feature space better aligned CD4/CD8 T cell lineage trajectories and suggested developmental origins of ILCs.

Our improved workflow revealed two unexpected data challenges and opportunities with scVDJ-seq. First, the high proportion of nonproductive TCR/BCR contigs suggests that these are unique challenges due to the choice of single-cell library construction. However, it is not unexpected as V(D)J rearrangement is a ‘wasteful’ exercise, a price to generate effective and diverse immune responses, for example, two of three rearrangement events for immunoglobulins are destined to be nonproductive^60,61^. While nonproductive and productive TCR/BCRs from high-throughput ‘bulk’ AIR sequencing data have been previously used together to estimate the generation probabilities and diversities of AIRs during affinity maturation and infection^62,63^, these would only have included contigs with *V* gene due to library limitations as discussed.

Second, detection of multi-J mapping suggests that these are naturally occurring and likely represent products of partial splicing events. While a few factors were identified to be associated with multi-J mapping, the biological implications are unclear at this stage, warranting future explorations.

We introduced a new way of analyzing the single-cell V(D)J modality in Dandelion with the pseudobulk V(D)J feature space, which can be used for visualization and differential V(D)J usage testing. When pseudobulked on cell neighborhoods, the V(D)J feature space is anchored to the underlying neighborhood gene expression space and can be used for pseudotime trajectory inference.

The first case study examined thymic T cell development. Previously, abT(entry) cells were suggested to be a divergent point as it was between DP T cells and mature single positive T cells^6^. With the V(D)J trajectory, we are now able to better delineate the branching point to a much earlier point within the abT(entry) cells. The new trajectories better aligned CD4/CD8 T cell fate with gene expression patterns of known marker genes and TFs and additionally revealed new associations with other TFs that remain to be explored.

This method can be useful for other applications for example studying T cell developmental stages across the lifespan, diseases and in vitro settings. It remains to be seen whether a VDJ-based trajectory can be used in T cell activation. This approach has not been optimized for BCR trajectories, as we are limited by the small number of B progenitors in available data. Further, BCRs have additional rearrangement rules that need to be considered, for example, somatic hypermutation, asymmetric usage of kappa/lambda light chains and light chain editing^64^, as well as recently described light chain coherence in functional antibodies. We hope to improve on these aspects in a future iteration of Dandelion when more single-cell V(D)J data become available.

The second case study extended the observations of nonproductive V(D)J contigs in single-cell data, which has been largely ignored and/or not easily accessible with other workflows, for example, scirpy^13^ and immcantation^23^. Our unexpected finding that B1 cells and pre–pro B cells expressed relatively higher levels of nonproductive TRB contigs suggest that B1 lineage commitment diverged earlier than expected, between the pre–pro B stage and pro-B stage. Two competing models have been described regarding B1 origin^65^. The lineage model or layered immune system hypothesis^66^ proposed that B1 and B2 cells arise from distinct progenitors that emerge at different times during development^67–70^, while the selection model hypothesized that they originate from the same progenitors but after differential signaling depending on self-reactivity^71,72^. Our findings here potentially offer a reconciliation of both models, with fetal-specific pre–pro B cells being B1 progenitors, supporting the layered immune system model, and the skipping of pre-BCR selection presumably allows the formation of self-reactive BCR, supporting the selection model.

Enrichment of the nonproductive TRB/TRG/TRD contigs was also observed in NK/ILC lineages. Partial recombination of TCR has been reported in mouse ILC^50,51^ and our findings support the ‘abandoned’ DN theory^52^. The hypothesis is that ILC/NK cells are originally on a canonical T cell development trajectory but subsequently influenced to abort this process, resulting in sustained expression of nonproductive TCR, although we cannot rule out other routes of ILC/NK development.

In summary, Dandelion is a freely available package for integrative analyses of single-cell GEX and V(D)J data. The V(D)J trajectories are also publicly available for use as a reference to a project or to align new query data. We hope that the software and resource will be useful in generating more insights into immune cell development and function.

## Methods

### Dandelion

#### Preprocessing

Dandelion can run the preprocessing of data using the standard outputs from all cellranger vdj versions. In this manuscript, single-cell V(D)J data from the 5′ Chromium 10X kit were initially processed with cellranger vdj pipeline (v6.1.2) with cellranger vdj reference (v5.0.0). TCR and BCR contigs contained in ‘all_contigs.fasta’ and ‘all_contig_annotations.csv’ from all three library types (αβTCR, γδTCR and BCR) were then re-annotated using an immcantation-inspired^23^ preprocessing pipeline contained in the Dandelion singularity container (v0.3.0).

The preprocessing pipeline includes the following steps:

i. Adjust cell and contig barcodes by adding user-supplied suffixes and/or prefixes to ensure that there are no overlapping barcodes between samples.
ii. Optionally subset to contigs deemed high confidence in the cellranger output; this was done in the analysis performed here.
iii. Re-annotation of contigs with igblastn (v1.19.0) against IMGT (international ImMunoGeneTics) reference sequences (last downloaded: 01/08/2021) with the following parameters: minimum *D* gene nucleotide match = 9, *V* gene *e*-value cutoff = 10^−4^; rearrangements missing the CDR3/junction sequences are enforced to be nonproductive (productive = ‘F’) and incomplete (complete_vdj = ‘F’).
iv. Re-annotation of *D* and *J* genes separately using blastn with similar parameters as per igblastn^25^ (dust = ‘no’, word size (J = 7; D = 9)) but with an additional *e*-value cutoff (J = 10^−4^ in contrast to igblastn’s default cutoff of 10; D = 10^−3^). This is to enable the annotation of contigs without the *V* gene present.
v. Identification and recovery of nonoverlapping individual *J* gene segments (under associated ‘j_chain_multimapper’ columns). In the list of all mapped *J* genes (all_contig_j_blast.tsv) from blastn, the *J* gene with the highest score (j_support) was chosen. Dandelion then looks for the next *J* gene with the highest ‘j_support’ value, and with start (j_sequence_start) and end (j_sequence_end) positions not overlapping with the selected *J* gene, and does so iteratively until the list of all mapped *J* genes are exhausted. In contigs without *V* gene annotations, we then select the 5′ end leftmost *J* gene and update the ‘j_call’ column in the final AIRR table. For contigs with *V* gene annotations, but with multiple *J* gene calls, we use the annotations provided by igblastn (NCBI IgBLAST Release 1.19.0’s release notes state that they ‘*Added logic to handle the case where there is an unrearranged *J* gene downstream of the VDJ rearrangement.’).

For BCRs, there are two additional steps:

i. Additional re-annotation of heavy-chain constant (C) region calls using blastn (v2.13.0+) against curated sequences from CH1 regions of respective isotype class.
ii. Heavy chain *V* gene allele correction using TIgGER (v1.0.0) (ref. ^74^). The final outputs are then parsed into AIRR format with change-o scripts^23^.

All the outputs from each step are saved in a subfolder, which the user can elect to retain or remove as per their requirements. Typically, a user would proceed with the file ending with the suffix ‘_contig_dandelion.tsv’ as this represents the rearrangement sequences that pass standard quality control checks. In this manuscript, we used the data found in the ‘all_contig_db-all.tsv’ as it also contains the multi-J mapping.

#### Postprocessing

In addition to the preprocessing steps at the contig level, postprocessing or integrating cell-level quality control is performed using Dandelion’s ‘check_contig’ function. The function checks whether a rearrangement is annotated with consistent *V*, *D*, *J* and *C* gene calls and performs special operations when a cell has multiple contigs. All contigs in a cell are sorted according to the unique molecular identifier (UMI) count in descending order, and productive contigs are ordered higher than nonproductive contigs. For cells with other than one pair of productive contigs (one VDJ and one VJ), the function will assess if the cell is to be flagged with having orphan (no paired VDJ or VJ chain), extra pair(s) or ambiguous (biologically irreconcilable, for example, both TCRs and BCRs in the same cell) status with some exceptions as follows: (1) IgM and IgD are allowed to coexist in the same B cell if no other isotypes are detected; (2) TRD and TRB contigs are allowed in the same cell because rearrangement of TRB and TRD loci happens at the same time during development, and TRD variable region genes exhibit allelic inclusion^75^. The function also asserts a library type restriction with the rationale that the choice of the library type should mean that the primers used would most likely amplify only relevant sequences to a particular locus. Therefore, if there are any annotations to unexpected loci, these contigs likely represent artifacts and will be filtered away. A more stringent version of ‘check_contigs’ is implemented in a separate function, ‘filter_contigs’, which only considers productive VDJ contigs, asserts a single cell should only have one VDJ and one VJ pair, or only an orphan VDJ chain, and explicitly removes contigs that fail these checks (with the same exceptions for IgM/IgD and TRB/TRD as per above). If a single-cell gene expression object (AnnData) is provided to the functions, it will also remove contigs that do not match to any cell barcodes in the gene expression data. Lastly, Dandelion can accept any AIRR-formatted data format, for example, BDRhapsody VDJ data.

#### Clonotype definition and diversity

Dandelion’s mode of clonotype definition and network-based diversity analysis has been previously described^4^. Briefly, TCRs and BCRs are grouped into clones/clonotypes based on the following sequential criteria that apply to both heavy-chain and light-chain contigs as follows: (1) identical *V* and *J* genes usage; (2) identical junctional CDR3 amino acid length and (3) CDR3 sequence similarity—for TCRs, 100% nucleotide sequence identity at the CDR3 junction is recommended while the default setting for BCRs is to use 85% amino acid sequence similarity (based on Hamming distance). Single-cell V(D)J networks are constructed using adjacency matrices computed from pairwise Levenshtein distance of the full amino acid sequence alignment for TCR/BCR(s) on a per-cell basis. A minimum-spanning tree is then constructed on the adjacency matrix for each clone/clonotype, creating a simple graph with edges indicating the shortest total edit distance between a cell and its neighbor. Cells with a total pairwise edit distance of zero are then connected to the graph to recover edges trimmed off during the minimum-spanning-tree construction step. A graph layout is then computed either using the Fruchterman–Reingold algorithm in networkx (≥v2.5) or Scalable Force-Directed Placement algorithm implemented through graph-tool (v2.46) package^76,77^. Visualization of the resulting single-cell V(D)J network is achieved via the transfer of the graph to relevant ‘AnnData’ slots, allowing for access to plotting tools in scanpy. The resulting V(D)J network enables computation of Gini coefficients based on cluster/cell size/centrality distributions, as discussed previously^4^.

#### Pseudobulk V(D)J feature space

Pseudobulk construction requires pseudobulk assignment information of cells, along with *V* and *J* genes for the cells’ identified primary TCR/BCR contigs (selected based on productive status and highest UMI count). The former is a cell by pseudobulk binary matrix, which can be either explicitly provided by the user or inferred from unique combinations of cell-level discrete metadata. While the code is calibrated to work with Dandelion’s structuring by default, it can work with any V(D)J processing provided it stores cell-level information on primary per-locus V/D/J calls. The input is used to generate a pseudobulk by V(D)J feature space, with the V(D)J calls converted to a binary matrix, added up for each pseudobulk, and normalized to a unit sum on a per-pseudobulk, per-locus, per-segment basis. The cell by pseudobulk information is stored in the resulting object for potential communication with the original cell space. Utility functions are provided for compatibility with Palantir^32^ output for trajectory inference.

### Nonproductive TCR/BCR contigs

Single-cell BCR, αβTCR and γδTCR data from ref. ^3^ were remapped with cellranger vdj (v6.1.2) and processed further using Dandelion as described above. For all samples, contigs were extracted from ‘all_contig_igblast_db-all.tsv’ or in the case whereby ‘all_contig_igblast_db-all.tsv’ was empty, ‘all_contig_igblast_db-fail.tsv’ was used. Preprocessed and annotated scRNA-seq data was downloaded from https://developmental.cellatlas.io/fetal-immune. Only contigs from annotated cells were kept for downstream analysis. For each contig, productive status was obtained from the column ‘productive’, and the causes for nonproductive contigs were extracted from ‘vj_in_frame’ (is ‘F’ if there is a frameshift), ‘stop_codon’ (is ‘T’ if there is a premature stop codon) and ‘v_gene_present’ (is ‘False’ if *V* gene is absent) columns.

### Cycloheximide treatment on PBMC

A vial of frozen PBMCs was acquired from Stemcell Technologies (70025.1) with informed consent (as stated by Stemcell Technologies) and approval from the Yorkshire & The Humber—Leeds East Research Ethics Committee (19/YH/0441). Frozen PBMCs were thawed in prewarmed RF10 media, which was RPMI (Corning, 10-041-CV) supplemented with 10% FBS (Gibco, A4766801) and penicillin/streptomycin (Sigma-Aldrich, P4333). Cells were pelleted by centrifugation at 500*g* for 5 min and resuspended in RF10 media, and split between two 10 cm Petri dishes. Control PBMCs were then incubated in a total of 10 ml RF10 media at 37 °C for 2 h, whereas treated PBMCs were incubated in RF10 supplemented with cycloheximide (Sigma-Aldrich, C4859-1ML; final concentration of 100 μg ml^−1^). After incubation, control and treated PBMCs were washed with ice-cold RF10 and resuspended in 2% FBS in PBS (Gibco, 14190144). For treated PBMCs, both the washing and resuspension buffer contained 100 μg ml^−1^ cycloheximide.

Control and treated PBMCs were then loaded onto two separate channels of the chromium chip from chromium single cell V(D)J kit (10X Genomics 5′ v2 PN-1000263) following the manufacturer’s instructions before droplet encapsulation on the Chromium controller. Single-cell cDNA synthesis, amplification, gene expression (GEX) and targeted BCR and αβTCR libraries were generated. Sequencing was performed on the Illumina Novaseq 6000 system. The gene expression libraries were sequenced at a target depth of 50,000 reads per cell using the following parameters: Read1, 26 cycles; i7, 8 cycles; i5, 0 cycles and Read2, 91 cycles to generate 75-bp paired-end reads. BCR and TCR libraries were sequenced at a target depth of 5,000 reads per cell.

Raw scRNA-seq reads were mapped with cellranger 3.0.2 with Ensembl 93-based GRCh38 reference. Low-quality cells were filtered out (minimum number of reads >2,000, minimum number of genes >500, maximum number of genes <7,000, maximum mitochondrial reads fraction <0.2 and maximum scrublet^78^ (v0.2.1) doublet score ≤ 0.5). Data normalization and log transformation were performed using scanpy^14^ (v1.9.1) (scanpy.pp.normalize_per_cell(counts_per_cell_after=10e4) and scanpy.pp.log1p). Highly variable genes were then selected (scanpy.pp.highly_variable_genes), and PCA (scanpy.pp.pca), neighborhood graph (scanpy.pp.neighbors) and UMAP (scanpy.tl.umap) were computed. Automatic annotation was done using celltypist (v1.2.0) (celltypist.annotate(model = 'Immune_All_Low.pkl', majority_voting = True)).

Single-cell αβTCR and BCR sequencing data were mapped with cellranger vdj (v6.1.2) and processed further using Dandelion, as described above. For all samples, contigs were extracted from ‘all_contig_igblast_db-all.tsv’ or in the case whereby ‘all_contig_igblast_db-all.tsv’ was empty, ‘all_contig_igblast_db-fail.tsv’ was used. Only contigs from annotated cells were kept for downstream analysis.

### Factors associated with multi-J mapping

#### Logistic regression analysis

We used the following logistic regression model to look for factors associated with multi-J mapping:

$$\mathrm{log}\frac{{p_i}}{{1 - p_i}} = \beta _{\mathrm{cell},c(i)} + \beta _{J,j(i)} + \beta _Vx_{V,i} + \beta _{{{\mathrm{cyclo}}}}x_{V,i}x_{{{\mathrm{cyclo}}},i}$$

where *p*_*i*_ is the probability of multi-J mapping present in the *i*th contig, *c(i)* and *j(i)* are the cell type and the 5′ end *J* gene of the *i*th contig, respectively, *x*_*V*,*i*_ is the indicator of whether *V* gene is present in the *i*th contig and *x*_cyclo,*i*_ is the indicator of whether *i*th contig belongs to a cell that had cycloheximide treatment. Here $\left( {\beta _{\mathrm{cell},c}:c \in \mathrm{cell}\;\mathrm{types}} \right)$, $\left( {\beta _{\mathrm{cell},j}:j \in 5{\prime}\;\mathrm{end}\;J\;\mathrm{genes}} \right)$, *β*_*V*_ and *β*_cyclo_ are parameters to be estimated.

To control for multiple testing, two-tailed *P* values were adjusted with BH procedure^79^. This was applied on all contigs from the γδTCR, αβTCR and BCR sequencing data that were identified within high-quality annotated cells from ref. ^3^ and results are shown in Supplementary Table 2, and it was also applied on contigs from the αβTCR and BCR sequencing data that were identified within high-quality annotated cells from control/cycloheximide-treated PBMCs and results are shown in Supplementary Table 3.

#### Splicing site motif analysis

For the lists of 5′ end *J* genes that had significant (BH adjusted two-tailed *P* < 0.05) association with increased or decreased multi-J mapping from Supplementary Table 2, the sequences of the last 11 nucleotides at each gene’s 3′ ends with the first ten nucleotides of its 3′ end intron were extracted from the 10X GRCh38 2020-A reference. Sequence logos shown in Fig. 2e were generated on https://weblogo.berkeley.edu/logo.cgi (ref. ^80^).

### γδTCR annotation comparison

To compare our γδTCR annotations against the 10X cellranger vdj output in the 33 γδTCR libraries^3^, we performed two additional mappings following 10X γδTCR support instructions. In one, the 5.0.0 reference was modified according to 10X instructions by replacing all instances of TRG with TRA and TRD with TRB. The reference was filtered to just TRG/TRD sequences before this replacement to avoid erroneous sequence overlaps. For the other, we performed the alignment with cellranger v7.0.0 with the accompanying reference (v7.0.0). The output of these two mappings was compared with the cellranger–Dandelion preprocessing pipeline described above. The number of high confidence γδTCR contigs and high confidence productive γδTCR contigs were determined for each mapping and each sample, and mappings were compared with the Wilcoxon signed-rank test. The effect size *r* is the rank correlation, which is the signed-rank test statistic divided by the total rank-sum^81^.

### Differential V(D)J usage in adult T cell subsets

Preprocessed and annotated scRNA-seq data of TLC and ILCs with paired αβTCR information from ref. ^5^ was downloaded from https://www.tissueimmunecellatlas.org/. Only cells within the T cell subsets with paired αβTCR were included in the downstream analysis. T_CD4/CD8 was excluded as a low-quality cell cluster. The cells were then pseudobulked by donor ID and cell type, and the pseudobulk V(D)J feature space was created with TRAV, TRAJ, TRBV and TRBJ. Only pseudobulks with at least ten cells were kept. PCA, neighborhood graph and UMAP of the pseudobulk V(D)J feature space were computed using scanpy^14^ (v1.9.1) with default settings (scanpy.pp.pca, scanpy.pp.neighbors, scanpy.tl.umap).

For low-level cell type annotations, Tem/emra_CD8, Tnaive/CM_CD8, Trm/em_CD8 and Trm_gut_CD8 were grouped into CD8^+^ T, and Teffector/EM_CD4, Tfh, Tnaive/CM_CD4, Tnaive/CM_CD4_activated, Tregs and Trm_Th1/Th17 were grouped into CD4^+^ T, while MAIT was left as a separate annotation. For differential V(D)J usage, Wilcoxon rank-sum test was performed using scanpy.tl.rank_genes_groups(method=‘wilcoxon’).

### Pseudotime inference from DP to mature T cells

#### Data integration and filtering

scRNA-seq data of human fetal lymphoid cells from ref. ^3^ was integrated with Dandelion preprocessed αβTCR, BCR and γδTCR data (see section ‘Nonproductive TCR/BCR contigs’, using all_contig_igblast_db-all.tsv for all samples) with dandelion.tl.transfer. Two samples from F67, F67_TH_CD137_FCAImmP7851896 and F67_TH_MAIT_FCAImmP7851897 were excluded from the analysis as they were sorted for specific T cell subpopulations, instead of the CD45 sorting in all other donor samples, and inclusion might result in biased TCR sampling within this donor. Only DP(P)_T, DP(Q)_T, ABT(ENTRY), CD8^+^ T and CD4^+^ T cells with productive TRA and TRB were included in the trajectory analysis. Neighborhood graph (scanpy.pp.neighbors(n_neighbors=50)) and UMAP (scanpy.tl.umap) were recalculated using scVI latent factors as the initial data was integrated with scVI^82^.

#### Pseudotime inference from neighborhood V(D)J feature space

Neighborhoods were sampled using Milo^30^ (milopy v0.1.0) (milo.make_nhoods). Cells were pseudobulked by the sampled neighborhoods and the V(D)J feature space was created with cells’ primary TRAV, TRAJ, TRBV and TRBJ genes. The cell type annotation of each neighborhood was assigned to be the most frequent annotation of the cells within that neighborhood. PCA, neighborhood graph and UMAP of the neighborhood V(D)J feature space were computed using scanpy^14^ (v1.9.1) with default settings (scanpy.pp.pca, scanpy.pp.neighbors, scanpy.tl.umap).

For pseudotime trajectory analysis, Palantir^32^ (v1.0.1) was used and diffusion map was computed using the first five principal components (PCs; (palantir.utils.run_diffusion_maps(n_components=5), palantir.utils.determine_multiscale_space). The root cell was chosen to be the DP(P) T neighborhood with the smallest value on UMAP1 axis, and the two terminal states were chosen with the largest and smallest values on the UMAP2 axis for CD4^+^ T and CD8^+^ T neighborhoods, respectively (Extended Data Fig. 3d). Pseudotime and branch probabilities to the terminal states were then computed with palantir.core.run_palantir(num_waypoints = *500)*.

Imputed pseudotime and branch probabilities were then projected back from neighborhoods (Fig. 3c) to cells (Fig. 4a, top panel) by averaging the parameters from all neighborhoods a given cell belongs to, weighted by the inverse of the neighborhood size. Cells that did not belong to any neighborhood were removed (91 of 17,248).

For pseudotime inferred with other trajectory inference methods as shown in Extended Data Fig. 7, monocle3 (ref*.* ^44^; 0.2.3.0) was applied on the UMAP embedding of the neighborhood V(D)J feature space and diffusion pseudotime^45^ was applied using scanpy.tl.dpt function with default settings. The same root cell neighborhood was used as above.

#### Pseudotime inference from neighborhood GEX feature space

Raw gene counts from scRNA-seq data were pseudobulked by the same cell neighborhoods as above. Data normalization and log transformation were performed using scanpy^14^ (v1.9.1) (scanpy.pp.normalize_per_cell(counts_per_cell_after=10e4) and scanpy.pp.log1p). Highly variable genes were then selected (scanpy.pp.highly_variable_genes), and PCA (scanpy.pp.pca), neighborhood graph (scanpy.pp.neighbors) and UMAP (scanpy.tl.umap) of the neighborhood GEX feature space were computed. Pseudotime trajectory inference was done similar to above with the first five PCs. The root cell was chosen to be the DP(P) T neighborhood with the smallest value on UMAP1 axis, and the two terminal states were chosen with the smallest and largest values on the UMAP2 axis for CD4^+^ T and CD8^+^ T neighborhoods, respectively (Extended Data Fig. 5c). Imputed pseudotime and branch probabilities were then projected back from neighborhoods (Extended Data Fig. 5d) to cells (Fig. 4a, bottom right panel).

#### Pseudotime inference from single-cell GEX

Pseudotime trajectory inference was performed with Palantir^32^ (v1.0.1) using the first 20 scVI latent factors. The root cell was chosen to be the DP(P) T cell with the largest value on UMAP2 axis, and the two terminal states were chosen with the largest value on the UMAP2 axis for CD8+ T and the smallest value on the UMAP1 axis for CD4^+^ T cells, respectively (Extended Data Fig. 5a). Results of the inferred pseudotime and branch probabilities are shown in Extended Data Fig. 5b.

#### Correlation between pseudotime ordering and relative TRAV/TRAJ locations

The relative genomic location of each TRAV gene was encoded numerically based on its order among all TRAV genes from 5′ to 3′ on the genome, and similarly for TRAJ. For each neighborhood, its relative TRAV or TRAJ location was computed by the average relative locations of all cells within that neighborhood. Only neighborhoods that had more than 90% cells being DP(Q) T cells were selected. The relative pseudotime order was plotted against the average relative TRAV or TRAJ location for each neighborhood in Fig. 4b. Local Pearson’s correlations were then computed over sliding windows of 30 adjacent neighborhoods on the pseudotime order (Extended Data Fig. 6a,b).

#### Correlation between gene expression and branch probabilities to CD8^+^ T in abT(entry) cells

Pearson’s correlations were computed between gene expression and branch probabilities to CD8^+^ T lineage within abT(entry) cells for all genes. Two-tailed *P* values were adjusted for multiple testing with BH procedure. Results are shown in Fig. 4c, Extended Data Fig. 6d and Supplementary Table 6.

### VDJ-based dimensionality reduction with CoNGA

Preprocessed and annotated scRNA-seq data of human fetal lymphoid cells from ref. ^3^ was downloaded from https://developmental.cellatlas.io/fetal-immune. Matching αβTCR samples had their all_contig_annotations.csv cellranger output files flagged with the sample IDs for both cell and contig IDs and were subsequently merged into a single file and subset to just high confidence contigs for cells present in the scRNA-seq object. This file was used on input for *CoNGA*’s^21^ (v0.1.1) setup_10x_for_conga.py script, which produced a tcrdist-based PCA representation of the cells’ VDJ data. The PCA coordinates were used to compute a neighborhood graph and UMAP representation (Extended Data Fig. 4a), using default scanpy settings.

### Joint embedding of single-cell gene expression and TCR with mvTCR

The same cells for which we performed pseudotime inference from DP to mature T cells above were used in the mvTCR^22^ (version under development, cloned from the repo at commit 528d3e11a360fc4b0f09d782b88f5ec7de9283d6) trial. Clonotypes were called based on CDR3 nucleotide sequence identity of the cells’ primary TRA and TRB chains (scirpy.pp.ir_dist, and scirpy.tl.define_clonotypes(receptor_arms=’all’, dual_ir=’primary_only’)).

Normalized and log-transformed data were used as recommended in mvTCR’s tutorial. The donor ID was one-hot encoded and supplied as a conditional variable. Eighty percent of cells were used as training data, the remaining 20% for validation. The models were trained for 200 epochs. Three runs were performed with the GEX to TCR ratio varying between 1:1, 2:1 and 3:1. Each run produced 15 trials and each trial had a different combination of model hyperparameters resulting from an automated hyperparameter grid search. The ‘best’ trial (lowest validation loss) was indicated at the end of each run; however, when we manually inspected all the trial results, we found the ‘best’ trials showed strong variations between different donors. Thus, we selected one representative result from each run with minimal cross-donor batch effects for Extended Data Fig. 4b.

### Pseudotime inference combining ILC/NK and T cells

#### Pseudotime inference using TRBJ

scRNA-seq data of human fetal lymphoid cells from ref. ^3^ was integrated with αβTCR data as described above. Only DN(early)_T, DN(P)_T, DN(Q)_T, DP(P)_T, DP(Q)_T, ILC2, ILC3, CYCLING_ILC, NK and CYCLING_NK cells with TRBJ were included for the trajectory analysis. Neighborhood graph (*k* = 50) and UMAP were recalculated using scVI latent factors similar to those above.

For pseudotime trajectory analysis, Palantir^32^ (v1.0.1) was used and a diffusion map was computed using the first five PCs. The root cell was chosen to be the neighborhood with the highest CD34 expression, and the two terminal states were chosen with the largest and smallest values on the UMAP1 axis for T and NK/ILC cell neighborhoods, respectively (Extended Data Fig. 10a). Pseudotime and branch probabilities to the terminal states were then computed and projected back from neighborhoods (Fig. 5b) to cells (Fig. 5c top panel).

#### Gene expression trend in DN T cells along pseudotime

Chatterjee’s correlations^73^ were computed between gene expression and inferred pseudotime within DN T cells for all genes that were expressed in at least 50 cells. Chatterjee’s correlation was chosen instead of Pearson’s or Spearman’s correlation to look for any functional change and not restricted to a monotonic change. TFs^83^ and genes encoding cell surface proteins that had significantly high Chatterjee’s correlation with pseudotime (BH adjusted *P* < 0.05; correlation coefficient >0.1) were shown in Fig. 5c and Extended Data Fig. 10b, respectively.

### Other visualization

In general, results were plotted using *seaborn* (v0.11.1; python) or ggplot2 (v3.3.3; R). Other single-cell plots were plotted using scanpy (v1.9.1). Visualization of Milo neighborhood graphs was plotted in R using ggraph (v2.1.0) and igraph (v1.2.6). Correlation/volcano plots were plotted in R using ggplot2 (v3.3.3). dplyr (v1.0.5) was used for general data frame handling for plotting in R.

### Reporting summary

Further information on research design is available in the Nature Portfolio Reporting Summary linked to this article.

## Online content

Any methods, additional references, Nature Portfolio reporting summaries, source data, extended data, supplementary information, acknowledgements, peer review information; details of author contributions and competing interests; and statements of data and code availability are available at https://doi.org/10.1038/s41587-023-01734-7.

## Supplementary information


Supplementary InformationSupplementary Fig. 1 and Supplementary Note.
Reporting Summary
Supplementary Tables 1–6Table 1: Top ten *J* gene combinations with multi-J mapping for each locus in data from ref. ^3^, with the number of contigs containing each combination shown next to it. Table 2: Logistic regression results exploring factors associated with multi-J mapping presence in data from ref. ^3^. Table 3: Logistic regression results exploring factors associated with multi-J mapping presence in control and cycloheximide-treated PBMC data. Table 4: List of leftmost (5′ end) *J* genes that had significant association with increased or decreased multi-J mapping, together with the sequences of their last 10 nucleotides at 3′ ends and the first 11 nucleotides of its 3′ end intron. Table 5: Differential V(D)J usage across CD4^+^ T, CD8^+^ T, and MAIT cells in data from ref. ^5^. Table 6: Pearson’s correlation coefficients and BH adjusted *P* values of all genes with branch probabilities to CD8^+^ T lineage within abT(entry) cells. [cor_tcr] Pearson’s correlation coefficients for pseudotime inferred from neighborhood V(D)J space. [pval_tcr] Pearson’s correlation *P* values for pseudotime inferred from neighborhood V(D)J space. [adjp_tcr] *P* values from pval_tcr adjusted by BH procedure. [cor_gex] Pearson’s correlation coefficients for pseudotime inferred from neighborhood GEX space.


## Data Availability

Raw sequencing data for newly generated sequencing libraries have been deposited in ArrayExpress (accession number E-MTAB-12524). Other datasets used are available at https://developmental.cellatlas.io/fetal-immune and https://www.tissueimmunecellatlas.org/.

## Code availability

Dandelion is implemented as an open-source package in Python 3 (https://github.com/zktuong/dandelion) with tutorials available at https://sc-dandelion.readthedocs.io/en/latest/. The tool and workflow are also available through an interactive online Google Colab notebook at https://colab.research.google.com/github/zktuong/dandelion/blob/master/container/dandelion_singularity.ipynb. Code and data used to generate figures and perform analyses in the manuscript are available at https://github.com/zktuong/dandelion-demo-files/dandelion_manuscript.
