# Supplementary material for: Dandelion uses the single-cell adaptive immune receptor repertoire to explore lymphocyte developmental origins
Source: Nat Biotechnol. 2023 Apr 13;42(1):40–51. doi: 10.1038/s41587-023-01734-7 (PMC10791579; doi:10.1038/s41587-023-01734-7)
Supplement: Supplementary file 1 — Supplementary Fig. 1 and Supplementary Note. [file 41587_2023_1734_MOESM1_ESM.pdf]

nature biotechnology

Article https://doi.org/10.1038/s41587-023-01734-7

# Dandelion uses the single-cell adaptive immune receptor repertoire to explore lymphocyte developmental origins

In the format provided by the authors and unedited

## Supplementary Note 1

The 10X Genomics library kit and V(D)J analysis for TCR was tailored for αβTCR contigs and most cellranger versions had difficulties in annotating γδTCR contigs, a problem 10X was aware of and addressed with user-side workaround instructions. When *cellranger vdj* updated its contig annotation pipeline in version 3.1.0 (https://support.10xgenomics.com/single-cell-gene-expression/software/pipelines/3.1/release-notes), the priority was αβTCR and the software lost the ability to annotate γδTCR contigs. However, the same release saw the introduction of custom enrichment primer support, which is integral for proper γδTCR contig reconstruction. γδTCR contig reannotation was reintroduced on an experimental basis in version 7.0.0 (https://support.10xgenomics.com/single-cell-gene-expression/software/pipelines/7.0/release-notes). *cellranger vdj* versions between 3.1.0 and 6.1.2 can still reconstruct γδTCR contigs, but cannot natively annotate them.

10X Genomics was aware of the issue, but it was not a priority for them as γδTCR libraries require custom enrichment primers not part of their product line-up. Early support requests would direct users to the last legacy version, 3.0.2, that supported γδTCR annotation (https://github.com/10XGenomics/cellranger/issues/45). However, this was not an ideal solution due to the lack of custom enrichment primer support. 10X Genomics subsequently revised their recommended solution to a modification of the reference, wherein all TRG sequences would be renamed to TRA and TRD would be renamed to TRB. This advice used to be available at https://kb.10xgenomics.com/hc/en-us/articles/360015793931-Can-I-detect-T-cells-with-gamma-delta-chains-in-my-V-D-J-data- but this has since been overwritten by *cellranger multi* instructions for version 7.0.0.

## Supplementary Figure Legend

**Supplementary Fig. 1 | Non-productive BCR in pDC. a,b,** Boxplot of the proportion of cells with productive (blue) or non-productive (orange) BCR heavy chain (**a**) and light chain (**b**) in different fetal myeloid subsets. Each point represents a sample and data were taken from Suo et al. 2022[1]. Only samples with at least 20 cells are shown. Boxes capture the first to third quartiles and whisks span a further 1.5X interquartile range on each side of the box. **c,** Expression of genes involved in V(D)J rearrangement in pDCs and cycling pDCs. Data was taken from Suo et al. 2022[1].

## Supplementary Tables

**Supplementary Table 1: top_10_j_multimappers.csv (separate file)**
Top 10 J gene combinations with multi-J mapping for each locus in data from Suo et al. 2022[1], with the number of contigs containing each combination shown next to it.

**Supplementary Table 2: LR_results.csv (separate file)**
Logistic regression results exploring factors associated with multi-J mapping presence in data from Suo et al. 2022[1].

**Supplementary Table 3: LR_results_combined.csv (separate file)**
Logistic regression results exploring factors associated with multi-J mapping presence in control and cycloheximide-treated PBMC data.

**Supplementary Table 4: j_sequence_affect_j_multimapper.csv (separate file)**
List of leftmost (5′ end) J genes that had significant association with increased or decreased multi-J mapping, together with the sequences of their last 10 nucleotides at 3′ ends and the first 11 nucleotides of its 3′ end intron.

**Supplementary Table 5: panimmune_differential_VDJ.csv (separate file)**
Differential V(D)J usage across CD4+T, CD8+T, and MAIT cells in data from Conde et al. 2022[2].

**Supplementary Table 6: abtentry_cor_result.csv (separate file)**
Pearson's correlation coefficients and BH adjusted *P*-values of all genes with branch probabilities to CD8+T lineage within abT(entry) cells.
[cor_tcr] Pearson's correlation coefficients for pseudotime inferred from neighborhood V(D)J space
[pval_tcr] Pearson's correlation *P*-values for pseudotime inferred from neighborhood V(D)J space
[adjp_tcr] *P*-values from pval_tcr adjusted by BH procedure
[cor_gex] Pearson's correlation coefficients for pseudotime inferred from neighborhood GEX space

## Supplementary References


1. Suo, C. *et al.* Mapping the developing human immune system across organs. *Science* **376**, eabo0510 (2022).
2. Domínguez Conde, C. *et al.* Cross-tissue immune cell analysis reveals tissue-specific features in humans. *Science* **376**, eabl5197 (2022).

**Supplementary Figure**

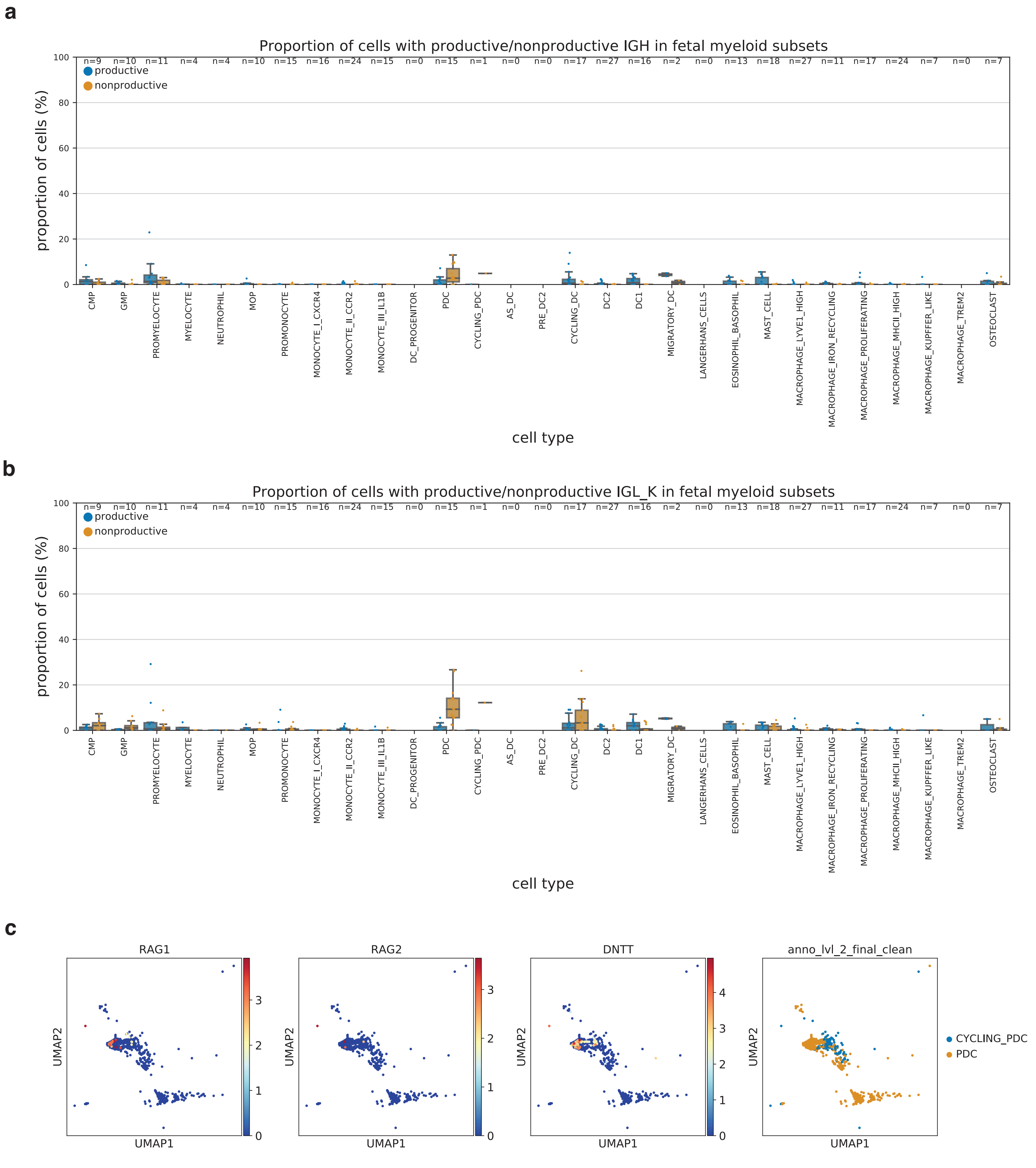


Suo et al. Supplementary Fig. 1
