# Supplementary material for: Dandelion uses the single-cell adaptive immune receptor repertoire to explore lymphocyte developmental origins
Source: Nat Biotechnol. 2023 Apr 13;42(1):40–51. doi: 10.1038/s41587-023-01734-7 (PMC10791579; doi:10.1038/s41587-023-01734-7)
Supplement: Supplementary file 2 — Reporting Summary [file 41587_2023_1734_MOESM2_ESM.pdf]

nature portfolio

Corresponding author(s): Zewen Kelvin Tuong, Menna R. Clatworthy, Sarah A. Teichmann

Last updated by author(s): Feb 28, 2023

Reporting Summary

Nature Portfolio wishes to improve the reproducibility of the work that we publish. This form provides structure for consistency and transparency in reporting. For further information on Nature Portfolio policies, see our Editorial Policies and the Editorial Policy Checklist.

Statistics

For all statistical analyses, confirm that the following items are present in the figure legend, table legend, main text, or Methods section.

n/a	Confirmed	
☐	☒	The exact sample size (*n*) for each experimental group/condition, given as a discrete number and unit of measurement
☒	☐	A statement on whether measurements were taken from distinct samples or whether the same sample was measured repeatedly
☐	☒	The statistical test(s) used AND whether they are one- or two-sided<br>*Only common tests should be described solely by name; describe more complex techniques in the Methods section.*
☐	☒	A description of all covariates tested
☐	☒	A description of any assumptions or corrections, such as tests of normality and adjustment for multiple comparisons
☐	☒	A full description of the statistical parameters including central tendency (e.g. means) or other basic estimates (e.g. regression coefficient) AND variation (e.g. standard deviation) or associated estimates of uncertainty (e.g. confidence intervals)
☐	☒	For null hypothesis testing, the test statistic (e.g. *F*, *t*, *r*) with confidence intervals, effect sizes, degrees of freedom and *P* value noted<br>*Give P values as exact values whenever suitable.*
☒	☐	For Bayesian analysis, information on the choice of priors and Markov chain Monte Carlo settings
☒	☐	For hierarchical and complex designs, identification of the appropriate level for tests and full reporting of outcomes
☐	☒	Estimates of effect sizes (e.g. Cohen's *d*, Pearson's *r*), indicating how they were calculated

Our web collection on statistics for biologists contains articles on many of the points above.

Software and code

Policy information about availability of computer code

Data collection: No software was used during data collection

Data analysis:
cellranger (v3.0.2)
cellranger vdj (v6.1.2 and v7.0.0)
igblastn (v1.19.0)
blastn (v2.13.0+)
tigger (v1.0.0)
conga (v0.1.1)

Python packages:
scanpy (v1.9.1)
celltypist (v1.2.0)
seaborn (0.11.1)
scrublet (v0.2.1)
palantir (v1.0.1)
milopy (v0.1.0)
graph-tool (v2.46)
dandelion: https://www.github.com/zktuong/dandelion

R packages:
dplyr (v1.0.5)

ggplot2 (v3.3.3)
igraph (v1.2.6)
ggraph (v2.1.0)

All other code and notebooks necessary to reproduce this manuscript can be found here in this github repository:
https://github.com/zktuong/dandelion-demo-files

For manuscripts utilizing custom algorithms or software that are central to the research but not yet described in published literature, software must be made available to editors and reviewers. We strongly encourage code deposition in a community repository (e.g. GitHub). See the Nature Portfolio guidelines for submitting code & software for further information.

Data

Policy information about availability of data

All manuscripts must include a data availability statement. This statement should provide the following information, where applicable:

- Accession codes, unique identifiers, or web links for publicly available datasets
- A description of any restrictions on data availability
- For clinical datasets or third party data, please ensure that the statement adheres to our policy

Dandelion is implemented as an open-source package in Python 3 (https://github.com/zktuong/dandelion) with tutorials available at https://sc-dandelion.readthedocs.io/en/latest/. The tool and workflow is also available through an interactive online Google Colab notebook at https://colab.research.google.com/github/zktuong/dandelion/blob/master/container/dandelion_singularity.ipynb. Code and data used to generate figures and perform analyses in the manuscript are available at https://github.com/zktuong/dandelion-demo-files/dandelion_manuscript. Raw sequencing data for newly generated sequencing libraries have been deposited in ArrayExpress (accession number E-MTAB-12524). Other datasets used are available at: https://developmental.cellatlas.io/fetal-immune and https://www.tissueimmunecellatlas.org/.

Human research participants

Policy information about studies involving human research participants and Sex and Gender in Research.

Reporting on sex and gender	Datasets were derived from published observational studies. The only new data was acquired from one sample of commercially available frozen peripheral blood mononuclear cells purchased from Stemcell Technologies. No information on sex or gender was collected.
Population characteristics	NA
Recruitment	NA
Ethics oversight	Frozen PBMCs (#70025.1) were acquired from Stemcell Technologies with informed consent (as stated by Stemcell Technologies) and approval from the Yorkshire & The Humber - Leeds East Research Ethics Committee (19/YH/0441).

Note that full information on the approval of the study protocol must also be provided in the manuscript.

Field-specific reporting

Please select the one below that is the best fit for your research. If you are not sure, read the appropriate sections before making your selection.

[x] Life sciences [ ] Behavioural & social sciences [ ] Ecological, evolutionary & environmental sciences

For a reference copy of the document with all sections, see nature.com/documents/nr-reporting-summary-flat.pdf

Life sciences study design

All studies must disclose on these points even when the disclosure is negative.

Sample size	No sample size calculations were performed. Sample sizes were determined based on the availability of datasets.
Data exclusions	No data were excluded.
Replication	Biological replicates were used as determined by the original studies where datasets were taken. We performed the cycloheximide experiments twice but the single-cell library from the first experiment failed. Due to budget limitations, we have only included the results from the second experiment.
Randomization	No randomization was performed as data were derived from observational studies.
Blinding	No blinding was performed for any analyses (not applicable to this paper). Datasets were derived from published observational studies.

Reporting for specific materials, systems and methods

We require information from authors about some types of materials, experimental systems and methods used in many studies. Here, indicate whether each material, system or method listed is relevant to your study. If you are not sure if a list item applies to your research, read the appropriate section before selecting a response.

Materials & experimental systems

n/a	Involved in the study
☒	☐ Antibodies
☒	☐ Eukaryotic cell lines
☒	☐ Palaeontology and archaeology
☒	☐ Animals and other organisms
☒	☐ Clinical data
☒	☐ Dual use research of concern

Methods

n/a	Involved in the study
☒	☐ ChIP-seq
☒	☐ Flow cytometry
☒	☐ MRI-based neuroimaging
